# Global Transcriptional Analysis Reveals Unique and Shared Responses in *Arabidopsis thaliana* Exposed to Combined Drought and Pathogen Stress

**DOI:** 10.3389/fpls.2016.00686

**Published:** 2016-05-24

**Authors:** Aarti Gupta, Ananda K. Sarkar, Muthappa Senthil-Kumar

**Affiliations:** National Institute of Plant Genome ResearchNew Delhi, India

**Keywords:** combined stress, *Pseudomonas syringae*, microarray, unique pathways, multiple stress tolerance, drought

## Abstract

With frequent fluctuations in global climate, plants are exposed to co-occurring drought and pathogen infection and this combination adversely affects plant survival. In the past, some studies indicated that morpho-physiological responses of plants to the combined stress are different from the individual stressed plants. However, interaction of drought stressed plants with pathogen has not been widely studied at molecular level. Such studies are important to understand the defense pathways that operate as part of combined stress tolerance mechanism. In this study, *Arabidopsis thaliana* was exposed to individual drought stress, *Pseudomonas syringae* pv tomato DC3000 (Pst DC3000) infection and their combination. Using Affymetrix WT gene 1.0 ST array, global transcriptome profiling of leaves under individual drought stress and pathogen infection was compared with their combination. The results obtained from pathway mapping (KAAS and MAPMAN) demonstrated the modulation in defense pathways in *A. thaliana* under drought and host pathogen Pst DC3000 infection. Further, our study revealed “tailored” responses under combined stress and the time of occurrence of each stress during their concurrence has shown differences in transcriptome profile. Our results from microarray and RT-qPCR revealed regulation of 20 novel genes uniquely during the stress interaction. This study indicates that plants exposed to concurrent drought and pathogen stress experience a new state of stress. Thus, under frequently changing climatic conditions, time of occurrence of each stress in the interaction defines the plant responses and should thus be studied explicitly.

## Introduction

Under field conditions drought stress most often occur in conjunction with pathogen infection and this combination negatively impact plant growth (McElrone et al., 2001; Mohr and Cahill, 2003; Choi et al., 2013). Drought potentially alters the plant-pathogen interaction by interfering with the host plant physiological and biochemical processes and making it either more, or less susceptible (Mohr and Cahill, 2003). Alternatively, drought-induced changes directly influence the pathogen survival in plant interface making it either more or less virulent (Wiese et al., 2004; Achuo et al., 2006; Goel et al., 2008; Hanso and Drenkhan, 2009; Luck et al., 2011). Furthermore, the combined effect of drought stress and pathogen infection leads to altered resistance responses (Xu et al., 2008; Choi et al., 2013; Prasch and Sonnewald, 2013). Previously, a few studies showed that stress interaction provoke altogether different transcriptome changes that were not seen under either of the individual stresses (Atkinson et al., 2013; Prasch and Sonnewald, 2013). Plant responses to combined drought and pathogen stress has been shown to vary depending on the severity and duration of each stress and also differs with the nature of infecting pathogens (Olson et al., 1990; McElrone and Forseth, 2004; Achuo et al., 2006; Xu et al., 2008). Till date, transcriptome changes in plants under combined drought and pathogen were documented in two studies (Choi et al., 2013; Prasch and Sonnewald, 2013). In the study on *Arabidopsis thaliana* subjected to drought and *Turnip mosaic virus* (TuMV) infection, transcriptome profiling revealed the existence of 24% of the shared genes between individual and combined stress while more than 50% of the unique genes were noted upon combined stress treatment (Prasch and Sonnewald, 2013). In another study, drought stressed *Vitis vinifera* showed enhanced disease symptoms under *Xylella fastidiosa* infection (Choi et al., 2013). Noteworthy, some of the tracheids infecting pathogens have been shown to cause physiological drought (McElrone et al., 2001, 2003). Hence, *X. fastidiosa* and drought stress have exerted synergistic impact on plant-water relations, and under such interaction 56% of differentially regulated genes were shared with either of the individual stresses (Choi et al., 2013). These studies indicate that the extent of shared responses between combined and individual stresses depends on the nature of pathogens that infect drought stressed plants. However, transcriptome changes in plants under drought and foliar pathogen has not been studied. Such studies are important to understand the genes and pathways that operate uniquely as part of combined stress tolerance mechanism. For plant-bacterial pathogen interaction studies in *A. thaliana, Pseudomonas syringae* is the best suitable system owing to its resource availability and well established methods (Katagiri et al., 2002). Both water deficit and foliar bacterial pathogen infection have been shown to modulate common plant responses. For example, drought induces stomata closure in the plants (Wilkinson and Davies, 2002), while some foliar bacterial pathogens force open the stomata (Melotto et al., 2008). Such changes thus can impact both this pathogen entry and regulate drought tolerance. In order to understand the modulation of defense reactions in *A. thaliana* under drought and *P. syringae* pv. tomato DC3000 infection, global transcriptome profiling of leaves under individual drought stress, pathogen infection and their combination was performed in this study. Transcriptome profile under different combined stresses was variably influenced by the time of occurrence of each stress during their concurrence. Our study also revealed unique responses under combined stress. Results from this study indicate that plants exposed to combined drought and pathogen stress experience a new state of stress that is different from plants exposed to the individual stresses. Thus, our transcriptomic study indicates the essential role of many unique genes under combined stress and further the existence of common genes between individual and combined stresses suggests a complex crosstalk between stress responsive mechanisms.

## Materials and methods

### Plant material, bacterial strain, and growth conditions

*Arabidopsis thaliana* ecotype Columbia-0 (Arabidopsis Biological Resource Center, accession number CS70000) was used in the study. Seeds were sown in agropeat (Prakruthi Agro Tech, Karnataka, India) and vermiculite (Keltech Energies Ltd, Maharashtra, India) mix (3:1 vol/vol, pre-weighed) and were stratified (for 48 h in dark at 4°C). Plants were grown under short-day conditions (8 h of light, 16 h of dark) with 200 μE m^−2^s^−1^ light intensity, 75% humidity, and 20°C constant temperature in growth chamber (PGR15, Conviron, Winnipeg, Canada). Plants were bottom irrigated with water or with Hoagland solution (Cat # TS1094, Himedia Laboratories, Mumbai, India) every alternate day till the start of the stress treatments.

*Pseudomonas syringae* pv. tomato DC3000 (Pst DC3000), a host pathogen of *A. thaliana* was used in this study. Pst DC3000 was grown in King's B medium (King et al., 1954) supplemented with rifampicin (50 μg/mL), at 28°C with continuous shaking of 200 rotations per minute (rpm) for 12 h.

### Preparation of Pst DC3000 inoculum

Bacterial culture at initial optical density at 600 nm (OD_600_) = 0.4 was centrifuged at 4270 g for 10 min. Bacterial pellet was washed thrice using sterile water and suspended in sterile water to the concentration of 5 × 10^3^ colony forming units (CFU)/mL.

### Combined stress imposition

Drought stress was initiated by withholding water from potted *A. thaliana* (32-days-old, 8 leaf stage). Gravimetric method (Ramegowda et al., 2013) was followed for stress imposition. Briefly, potted plants were weighed twice a day (11:00 a.m. and 5:00 p.m.) and were brought down to a defined level of drought stress, 40% soil field capacity (FC) (Ψw = −3.9 MPa). Control plants were maintained at 100% FC. According to previous standardizations, the potting mix used in the study attained 40% FC in 5 days (Supplementary Figure S1A). FC was determined using the following formula;

$$\begin{array}{l} \text{FC }\left(\%\right)=\left[\left(\text{WW }-\text{ DW}\right)∕\text{DW}\right]\times 100 \end{array}$$

WW-wet soil weight; DW-air dry soil weight.

Upon the arrival of 40% FC (37-days-old plants), pathogen was infiltrated at 5 × 10^3^ CFU/mL concentration through abaxial side of the leaves (DP). The time of pathogen inoculation was considered as 0 h post-treatment (hpt), such that plant experiences combined drought stress of 40% FC along with pathogen stress for 24 hpt. Individual drought stressed and pathogen infected plants were separately maintained. The control healthy plants were infiltrated only with sterile water (mock inoculation).

For pathogen first and concurrent drought later treatment, pathogen was infiltrated into the leaves (of 32-days-old–plants) and such plants were subjected to water withdrawal (PD). Individual plants maintained only at 40% FC and others treated only with pathogen were also maintained. The outline for combined stress protocol is provided in Supplementary Figure S1A.

### Assessment of *In planta* bacterial multiplication

Bacterial multiplication in leaves from combined stressed and pathogen only treated plants was assessed at 24 hpt. Circular discs measuring 1 cm diameter were cut out using cork borer from infected leaf. The leaf disc was surface sterilized with 0.01% H_2_O_2_ for 20 s, and was homogenized in 1000 μL of sterile water. Upon further serial dilution in sterile water, it was plated on King's B agar medium supplemented with rifampicin antibiotic (50 mg/L). Bacterial population was calculated as CFU/cm^2^ (Wang et al., 2007). Bacterial numbers were calculated as per the following formula:

(2) $$\begin{array}{l} \text{Bacterial multiplication }\left(\text{CFU}/{\text{cm}}^{2}\right)\text{=}\\ \;\frac{\frac{\begin{array}{l} \text{Number of colonies x volume of homogenate }(\mu \text{L})\\ \text{ x dilution factor} \end{array}}{\text{volume plated}}}{\text{Leaf area }({\text{cm}}^{2})} \end{array}$$

### Assessment of membrane leakage

Electrolyte leakage was measured in leaf samples following protocol described by Tripathy et al. (2000). Briefly, discs (1 cm diameter) were punched from each leaf and were rinsed in deionized water for 2 min to remove lysed contents due to cut ends. Washed leaf discs (two discs) were placed in 20 mL of deionized water for 12 h with continuous shaking at 60 rpm at 20°C. Six biological replicates were considered for each treatment. Conductivity of the bathing solution was measured for each sample using conductivity meter (Model-1602, EC-TDS-SAL Meter, Esico International, Himachal Pradesh, India). Samples with bathing solution were autoclaved to cause complete (100%) electrolyte leakage and conductivity was measured again. Electrolyte leakage was expressed as the percentage ratio of initial and final readings.

### RNA extraction

Leaf tissue (100 mg fresh weight) from third tier of stressed and unstressed rosette was harvested at 24 hpt and immediately frozen in liquid nitrogen. The frozen material was pulverized in liquid nitrogen into a fine powder. The homogenate was used for isolation of total RNA using RNeasy plant mini kit (Cat # 74904, Qiagen, Hilden, Germany) as per the manufacturer's instructions. RNA samples were treated with DNase I (Cat # 79254 RNeasy/QlAamp columns, Qiagen, Hilden, Germany) to remove DNA according to the manufacturer's instructions. RNA quality and quantity were evaluated using an Agilent 2100 Bioanalyzer (Agilent Technologies, California, USA) with RNA 6000 Nano Chips (Cat # 5067-1511, Agilent Technologies, California, USA), following the manufacturer's protocol. RNA integrity numbers (RIN) ranged from 7.0 to 7.5.

### Microarray hybridization

Microarray experiment was conducted using Whole Transcript (WT) Expression Arrays (Affymetrix, California, USA). Total RNA was labeled using GeneChip® WT PLUS Reagent Kit (Cat # 902281, Affymetrix, California, USA) as per manufacturer's protocol. Briefly, total RNA (500 ng) was reverse transcribed to synthesize single-stranded cDNA with T7 promoter sequence at the 5′ end. Template cDNA was converted to double-stranded cDNA, simultaneously degrading the residual RNA. Complimentary RNA (cRNA) was synthesized and amplified by *in-vitro* transcription of the second-stranded cDNA template using T7 RNA polymerase. cRNA was purified and reverse transcribed to make sense strand cDNA containing dUTP at a fixed ratio relative to dTTP. Template RNA was removed following hydrolysis using RNase H. The purified, sense-strand cDNA is fragmented at the dUTP residues and was labeled using Affymetrix proprietary DNA labeling reagent that is covalently linked to biotin. The appropriate amount of each fragmented and biotin-labeled single stranded (ss)-cDNA was mixed with hybridization master mix and was loaded onto cartridge (GeneChip® Gene 1.0 ST, Cat # 901915, Affymetrix, California, USA). Loaded cartridge was hybridized for 16 h at 45°C and 60 rpm followed by washing, staining, and scanning using GeneChip® hybridization, wash, and stain kit (Cat # 900720, Affymetrix, California, USA) according to the manufacturer's guidelines. Six different plants were maintained for each treatment and a pool of three plants were sampled for each biological replicate. For each treatment, two biological replicates were hybridized (Supplementary Figure S2A).

### Microarray data extraction and analysis

A concise protocol depicting data analysis is presented in Supplementary Figure S2A. In short, image files (.CEL) were imported into GeneSpring GX 12.1.6 (Agilent Technologies, California, USA). The microarray data was normalized using RMA algorithm (GeneSpring GX 12.1.6). Unpaired *t*-test was employed to obtain differentially expressed genes. More than 2-fold differentially expressed genes (DEGs), between two conditions (treatment over control) with *t*-test *p* ≤ 0.05 were selected for further analysis. The selected DEGs were segregated into up- and down-regulated genes. Up-regulated genes were assigned positive values, and down-regulated ones as negative values.

Genes were distributed along gene ontology (GO) biological classes using inbuilt feature of GeneSpring (Agilent GeneSpring GX12.1.6) and relative enrichment was performed as percentage of significantly regulated genes from a specific functional group relative to the total genes from that specific group to the entire chip. Genes were also categorized as per GO molecular function according to TAIR GO functional classification (TAIR 10). DEGs across individual and combined stress treatments were compared using Venn diagrams (Agilent GeneSpring GX12.1.6). Based on Venn intersections, DEGs were segregated as unique to combined stress transcriptome and as common between individual and combined stressed transcriptome.

The pathway networks were determined by input of the selected gene lists into KEGG Automatic Annotation Server (KAAS, http://www.genome.jp/tools/kaas/) and MAPMAN (http://mapman.gabipd.org/web/guest/mapman). Heat maps were drawn with fold change values using GENE-E software (http://www.broadinstitute.org/cancer/software/GENE-E/).

### Quantitative real-time PCR analysis

Transcript expression of selected genes was quantified by real-time PCR (RT-qPCR) using ABI Prism 7000 sequence detection system (Applied Biosystems, California, USA). For all the RT-qPCR experiments, three independent biological replicates were performed. Total RNA (5 μg) was reverse transcribed to make template (first strand cDNA) in a reaction volume of 50 μL using verso cDNA synthesis kit (Cat # AB1453A, Thermo Scientific, Massachusetts, USA). Gene-specific primers (Supplementary Table S1) were designed using Primer 3 software (Untergrasser et al., 2012). Template cDNA was diluted (5-fold) and was mixed with 750 nM each of the specific primers and SYBR Green PCR master mix (Applied Biosystems, USA) in a final volume of 10 μL. Ct values obtained for *AtACTIN2* (AT3G18780) gene was used to normalize data. Fold change in gene expression in stressed samples was quantified using comparative D cycle threshold (CT) method relative to the non-stressed control samples (Livak and Schmittgen, 2001).

### Statistical analysis

Data presented are average of biological replicates. The numbers of biological replicates considered for each experiment are mentioned in the legend for respective figure. Error bars represent ± SEM. Significant differences among treatments were determined by one-way ANOVA and applying the least-significant difference *post-hoc* Tukey's test (*p* < 0.05) (SigmaPlot 11.0, Systat Software Inc., California, USA). The test of significance applied in RT-qPCR analysis was Student's *t*-test (*p* < 0.05) (SigmaPlot 11.0, Systat Software Inc., California, USA).

## Results

### Combined stress imposition lead to drought and pathogen defined plant physio-morphological changes

Leaves of *Arabidopsis thaliana* were exposed to combined drought and bacterial pathogen (Pst DC3000) stress by following two different protocols. In the first protocol, drought stress was started first and then pathogen inoculation was done (hereafter referred as DP). Initially, plants were maintained at 100% field capacity (FC), which corresponds to ~92% leaf relative water content (RWC) and at the start of the experiment, water was withheld so that pots come down to 40% soil moisture content (FC) with leaves exhibiting ~55% RWC (Supplementary Figure S1). In the DP protocol, plants were allowed to experience gradual drought and the pathogen was inoculated at 40% field capacity (FC). Plants were maintained at this drought level along with continued *in vivo* pathogen multiplication for 24 h. Gravimetric assessment revealed that plants realized progressive drought at 80 and 60% FC for 1 and 2 days respectively before reaching to a final drought stress level of 40% FC (Supplementary Figure S1A). Individual stressed plants namely drought plants at 40% FC and pathogen inoculated plants along with absolute control and mock (water only) inoculated plants were maintained as controls and data gathered were compared with appropriate controls (Supplementary Figure S1A).

In the second protocol, pathogen was inoculated and then the irrigation was stopped to induce drought stress. *In planta* bacterial numbers at 0 hpt reflect delivery of 2.14 Log (CFU/cm^2^) concentration of pathogen in the inoculated leaf. Drought stress at 40% FC had reached 5 days after pathogen inoculation. During this period, both exponential pathogen multiplication and progressive drought stress were observed in these plants (Supplementary Figure S1). At 40% FC with progressive pathogen multiplication, plants were maintained for 24 h and samples were then taken for experiments. This protocol where pathogen was inoculated before start of drought stress is referred as PD. Individual stressed plants namely, drought stressed and pathogen infiltrated (for 6 days of prolonged duration, referred as PP) along with mock inoculated and absolute control plants were maintained in this experiment. Another control involving mock (water) infiltration into the leaves of drought stressed plants (DM) was also included for transcriptomic study. Raw and processed result files generated from microarray experiment were submitted to GEO NCBI (accession no. GSE79681). Results on RWC, *in planta* bacterial numbers and transcriptomic analysis from combined stressed plants indicated successful drought stress imposition and pathogen infection (Supplementary Figures S1C,D, S3). Results from transcriptome analysis revealed presence of drought and pathogen responsive genes examples, those encoding for late embryogenesis abundant proteins (LEAs), dehydrins, chitinases and pathogenesis related (PR) proteins (Welin et al., 1994; Katagiri et al., 2002; Zhao et al., 2003; Hanin et al., 2011; Yang et al., 2012), among the most differentially expressed transcripts in response to stress (Supplementary Figure S3). Further, the results on gene ontology biological process revealed that the top-most differentially expressed genes (DEGs) were enriched with GO biological process “response to stress” (Supplementary Figure S4).

Influence of combined stress on pathogen infection and disease progression was studied and we observed reduced *in planta* bacterial multiplication in DP stressed plants. However, PD stressed plants showed bacterial numbers similar to pathogen only inoculated plants. Accordingly, DP stressed plants did not show disease-induced chlorosis, but conspicuous chlorosis was observed in PD stressed plants (Supplementary Figure S1B). Also, the RWC in DP and PD stressed plants was 55 and 46% leaf water content respectively and no significant difference in RWC was found between DP and PD. Since independently occurring drought and pathogen induces leakage of solutes through cellular membranes, electrical conductivity measurement was considered as parameter for assessing stress impact in combined stressed plants. Membrane leakage was higher under combined DP stress (38%) in comparison to the drought (26%) or pathogen (22%) stresses. However, in case of the combined PD stress (33%), the leakage was at par with pathogen (PP) induced damage (34%) (Supplementary Figure S1E). All these results indirectly suggest that pathogen infection does not alter the drought stress specific impact on plants in both the combined stress protocols. In this manuscript, the changes in plant defense against pathogen influenced by drought stress are explored.

Our experiments revealed two major inferences. One, the first occurring individual stress likely plays role in deciding outcome of stress interaction. Based on the initial analysis of transcriptome data (Supplementary Figures 1A,B) we predicted existence of both unique and shared responses that can explain the observed physio-morphological changes. Second, the likely “dominant” stressor, drought, seem to play role in reducing the bacterial multiplication in DP plants, but not in PD plants. The transcriptomic data for DP and individual stress controls revealed changes in plant defense responses. Hence, we first present the analysis of the net effect of combined stresses (DP and PD) on plants. In second part of the manuscript, we present transcriptome analysis and provide reasons for reduction in bacterial numbers in DP or pathogen number in PD plants similar to pathogen infiltrated plants.

### Combined stressed plants showed distinct transcriptome changes

In order to understand the transcriptome changes in combined stressed plants, whole-transcripts expression arrays were performed using RNA samples isolated from plants (leaf tissue) exposed to individual and combined stress conditions (Supplementary Figure S2A). Hierarchical clustering based on expression values of the transcripts showed that the two replicates of differentially treated plants clustered together (Supplementary Figure S2B). Drought stressed plant infiltrated with water (drought-mock, DM) clustered closely to the drought stressed plants, inferring that the syringe infiltration method (water and wound response) used in this study are accommodated in our analysis so that the exclusive treatment effects are compared. Both the combined stress treatments (DP and PD) were close to the drought stress clade whereas transcriptome profile under pathogen and prolonged pathogen treatment formed a separate clade, possibly reflecting the dominance of drought stressor in the combined stress (Supplementary Figure S2B).

**Figure 1:**
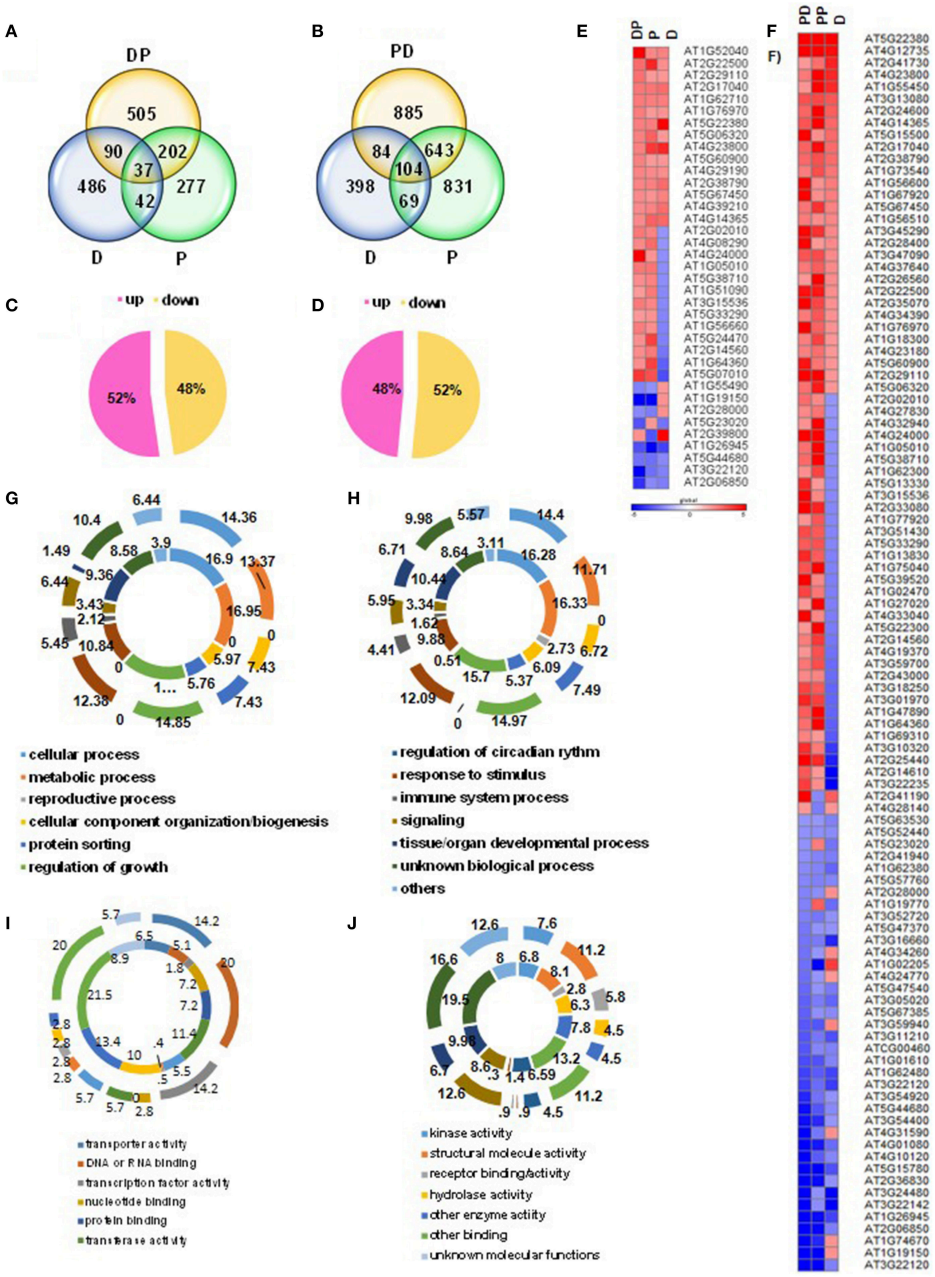
**Summary of comparative transcriptome profile of combined stressed plants with individual stressed plants**. *Arabidopsis thaliana* plants (32-days-old) were exposed to individual stresses, i.e., drought (D), *Pseudomonas syringae* pv. tomato DC3000 (Pst DC3000; P). Combined stress treatments involved superimposition of pathogen infection on drought stressed plants (DP) or drought imposition in already pathogen infected plants (PD). Microarray experiment was carried out using Affymetrix WT gene chip array and differentially expressed genes (DEGs) in each stress treatment were identified in comparison to control samples (fold change > 2, unpaired *t*-test *p* < 0.05). Venn intersection between DEGs in individual and combined stress highlights the unique and shared transcriptome profile in response to DP and PD combined stress **(A,B)**. Percent distribution of uniquely expressed genes in up- and down-regulated category in response to combined DP and PD stress treatments is shown here **(C,D)**. Expression pattern of genes shared among D, P, and DP; D, PP, and PD is depicted as heat map **(E,F)**. Color bar scale shows the fold change range with red and blue color representing up- and down-regulation respectively. Gene ontology (GO) biological function based functional categorization (Agilent GeneSpring GX12.1.6) was done for DEGs unique to combined stress and shared between D, P and DP **(G)** D, PP and PD **(H)**. GO molecular function based categorization (as per TAIR) was done for DEGs unique to combined stress and shared between D, P and DP **(I)** and D, PP and PD **(J)**. Outer ring represents the GO categories for genes shared between individual and combined stress treatments. Inner ring represents the genes unique to the combined stress treatments. Gene names and descriptions for the gene IDs presented in the figure are provided in Supplementary File S2.

We present DP and PD transcriptome changes under two major categories of responses. One is exclusive to combined stress, i.e., “unique” and the second encompasses common genes between individual and combined stresses that either have expression pattern similar to individual stress termed as “shared” or those with “tailored” expression pattern compared to individual stresses. Unpaired *t*-test listed out the significant and differentially regulated genes in each stress condition compared with respective controls. Transcriptome profile under each condition is listed in Supplementary File S1. Figures 1A,B illustrates the numbers of statistically significant differentially expressed transcripts under each stress condition (File S1). For instance, the number of differentially regulated transcripts in individual drought and pathogen stress and their combination were 655, 558, and 834 respectively. However, when pathogen was infiltrated ahead of drought stress (PD) imposition, there was a pronounced increase in the number of differentially regulated transcripts as 1647 and 1716 under prolonged pathogen (PP) and PD combined stress respectively.

Venn intersections depicted the unique and common transcriptional responses (or common) between individual and combined stress. Comparison of differentially expressed genes across individual and combined stress treatments revealed 37 genes common among drought, pathogen and DP treatments and 104 genes common among drought, prolonged pathogen and PD treatments. These common genes however showed shared or tailored expression between individual and combined stress. As presented in heat maps, 19 genes were shared and 18 genes were tailored in DP stress compared to the individual stresses (Figure 1C). Similarly 59 genes showed shared expression pattern among individual drought, prolonged pathogen and PD combined stress while, 45 genes showed tailored expression pattern between individual and combined stressed plants (Figure 1D). Our analysis revealed a substantial number of genes which were induced exclusively upon combined stress treatment (unique genes), were absent from the list of DEGs under individual drought or pathogen treatments. Combined DP stressed plants exhibited 505 unique DEGs and combined PD plants exhibited 885 numbers of unique DEGs. Strikingly, when the unique transcripts under DP stress were compared with that of unique PD, a large number of genes were found to be common between the two. Moreover, expression pattern of these genes under DP and PD stress was also similar (Supplementary Figure S5). Comparative expression profile of “stress category” genes from the list of total DEGs under combined DP and PD stress revealed ~35% of common stress genes. Moreover these common stress genes exhibited similar expression pattern under DP and PD stress (Supplementary Figure S8). This indicates that, in order to combat combined stress (involving drought and bacterial pathogen), possibly plants tend to modulate a certain number of basal combined stress responsive genes.

Few of the unique transcripts under DP stress (in comparison to D and P stress, based on gene IDs) belong to a gene family. The others members of such gene families were observed to be differentially expressed under individual drought or pathogen stress (File S2). The expression pattern of the different isoforms of a gene may depend on the nature of the stress. These included calcium-dependent lipid-binding domain-containing protein, leucine rich repeat (LRR) transmembrane protein, transcriptions factors from NAC, WRKY, and MYB families. The membrane localized LRR proteins have been implicated in sensing the external environment while members of these transcription factor families are involved in signal transduction and plant responses to various abiotic and biotic stresses (Jones and Dangl, 2006; Zheng et al., 2007; Dubos et al., 2010; Nuruzzaman et al., 2013; Osakabe et al., 2013; Bakshi and Oelmüller, 2014; Nakashima et al., 2014).

**Figure 2:**
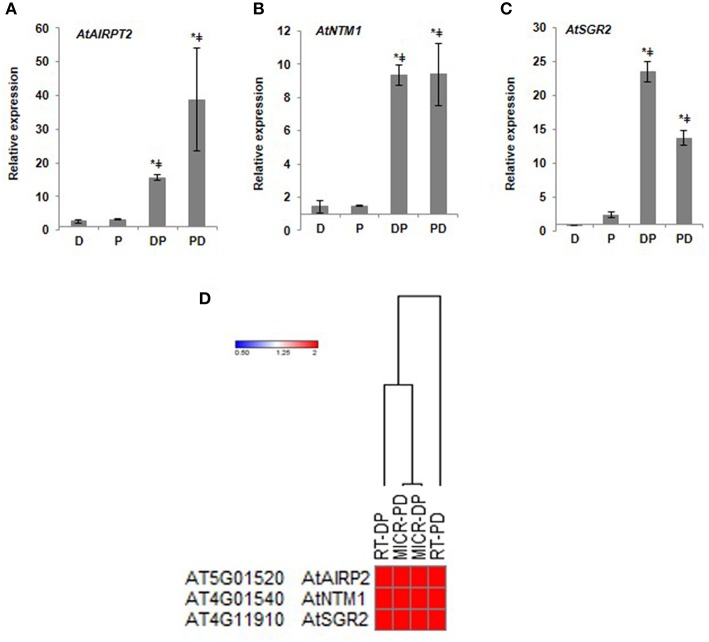
**RT-qPCR validation of microarray data from DP combined stressed leaves**. Relative expression of *AtAIRP2* **(A)** *AtNTM1* **(B)**, *AtSGR2* **(C)** genes is presented. Candidate genes were selected and corresponding transcript accumulation under combined stress treatment was quantified by RT-qPCR. Fold change in expression levels relative to the control samples were normalized to *AtACTIN2* gene expression. Each experiment was carried out with three biological and two technical replicates. Data represents the average of three biological replicates and error bars shows standard error of mean (SEM). Significance was calculated using Student's *t*-test where ^*^ and ^‡^ symbol shows significance at *p* < 0.05 over drought and pathogen stress respectively. Heat map depicts the correlation between microarray and qRT-PCR data. Dendrogram was based on Euclidean distance **(D)**. MICR, fold change values from microarray experiment; RT, fold change values from RT-qPCR analysis. Color bar in red and blue color represents up- and down-regulated genes respectively. Gene names and descriptions for the gene IDs presented in the figure are provided in Supplementary File S2.

To identify the key genes involved in combined stress responses, we analyzed expression pattern of 20 genes unique to combined DP stress through RT-qPCR. Few of these candidate genes belong to multigene family (other isoforms are implicated in individual stress tolerance, File S2). These genes include those encoding for ring finger protein, receptor like kinase, plant natriuretic peptide (PNP), sugar transporter, sodium hydrogen exchanger and nuclear transcription factor. For example, earlier literature indicated that PNPs are secreted into the apoplast and elicit a range of host defense responses such as tissue specific modifications of cation transport, changes in stomatal conductance and the photosynthetic rate (Turek et al., 2014). RT-qPCR results showed expression pattern similar to microarray for the tested genes indicating induction of these genes under combined stress (Figure 2; Supplementary Figure S7). Gene encoding ABA insensitive ring protein 2, a RING/U-box superfamily protein was up-regulated under both drought and pathogen stress but was induced to 15-folds upon combined DP stress. This gene has previously been shown to be involved in drought stress and pathogen infection (Fabro et al., 2008; Cho et al., 2011). Another uniquely expressed transcription factor was NAC with transmembrane motif1 (NTM1). As reflected in our RT-qPCR results the expression pattern of this gene was unique to combined stress and presence of transcripts under drought or pathogen only stress was observed only to the minimal levels. This is a membrane protein tethered to ER or nuclear membrane and possibly has a role in ER stress, like other members of this family (Kørner et al., 2015). Stay green 2 encoding a chlorophyll catabolic protein showed up-regulation under pathogen stress but under combined stress it was induced to 23-fold change. This could be plant's counter defense to pathogen induced chlorophyll degradation. Our result is further strengthened by the information curated from public (eFP browser) and literature data under drought stress or Pst DC3000 infection. The genes showing unaltered expression under drought or Pst DC3000 infection are more suitable candidates to be established as “unique genes” under combined stress and are likely to be involved in plant adaptations to combined stress (over individual stress).

### Unique and shared genes were specifically enriched for pathways contributing to defense and growth

Genes unique to the combined stress and genes common between individual and combined stress treatments were further classified to inferred biological process using the Gene Ontology (GO) terminologies provided by GeneSpring. Our results implicate that GO terms were similarly enriched with unique genes from DP and PD combined stress treatments (Figures 1E–I, Supplementary Figure S6; Supplementary Tables S2, S3). Likewise common genes were similarly enriched with GO terms under both kinds of combined stress treatments (Figures 1E–I; Supplementary Tables S2, S3). However, a comparison of unique vs. common genes hinted toward prioritized plant responses under combined stress for growth, signaling and immune system responses. For instance, the GO term “growth” was completely absent from the common genes and at the same time “signaling” term was less enriched with common genes in comparison to the unique genes under DP stress (Supplementary Table S3). Unique genes induced under DP and PD combined stress were associated with similar pathways (mapped through KAAS) viz., carbon, nitrogen, sulfur, fatty acid and amino acid metabolism, photosynthesis, secondary metabolites and wax biosynthesis. In spite of the common stressors involved, the two kinds of combined stresses, i.e., DP and PD invoked different pathways (Supplementary Tables S4, S5). For example, unique DEGs under DP stress mapped to inositol phosphate metabolism, thiamine metabolism, folate biosynthesis and ABC transporter while PD unique genes mapped to linoleic acid metabolism, glycan degradation and nucleic acid synthesis, repair, transport and degradation (Supplementary Tables S4, S5). DP combined stress activated diterpenoid biosynthesis while monoterpenoid biosynthesis was induced upon PD combined stress (Supplementary Tables S4, S5). Thus, the two different protocols followed for combined stress actually represented two different kinds of combined stress, possibly the time of stress decides the plant defense responses.

### Basal disease resistance pathway might contribute to drought-induced endurance to pathogen infection

**Figure 3:**
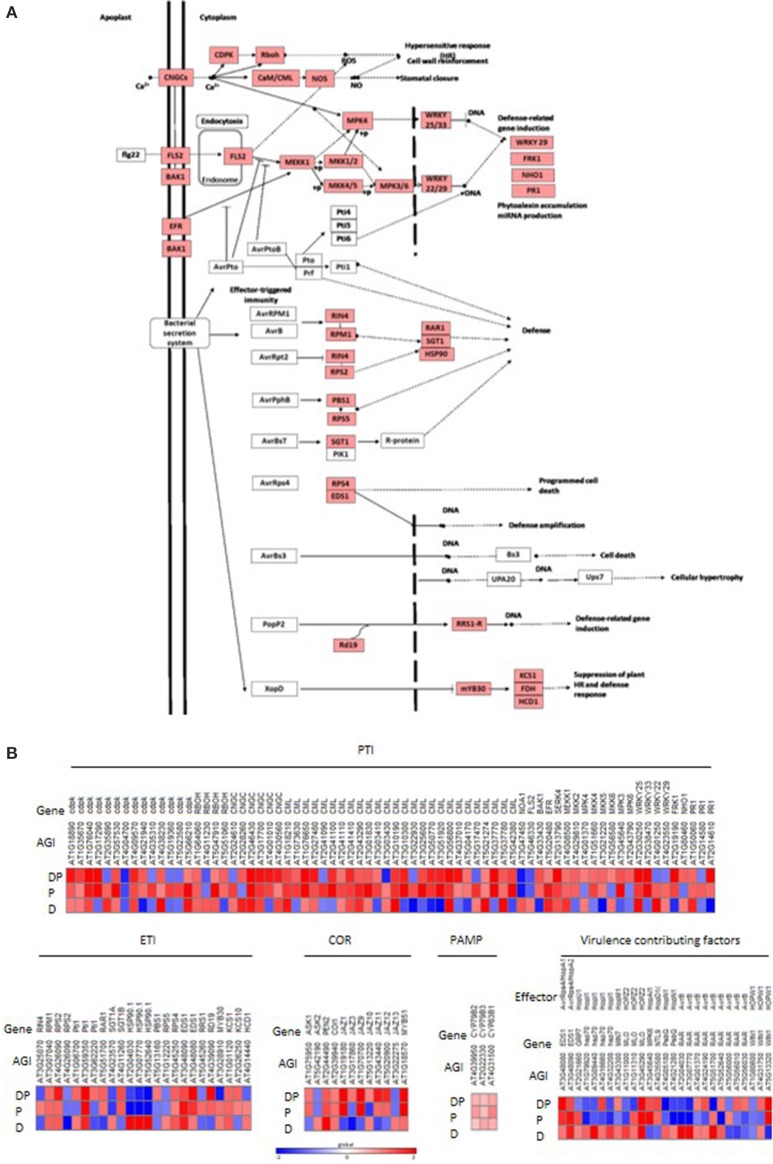
**Modulation in expression pattern of “defense” related transcripts under combined DP stress**. Illustration depicts bacterial PAMP and effector triggered pathways during plant-pathogen interaction (redrawn from KEGG) **(A)**. Heat map represents expression pattern of different genes involved in plant-pathogen interaction **(B)**. Differentially expressed genes were listed out using Affymetrix® Expression Console (EC) and Transcriptome Analysis Console (TAC). DEGs under DP combined stress were associated with PAMP triggered immunity (PTI) and effector triggered immunity (ETI), according to annotation provided by KAAS. Genes influenced by bacterial virulence factor coronatine (COR), PAMP or different bacterial effectors (secreted into the plant cell and contributing to the virulence) were manually curated. Fold change values were used to plot heat map where color bar scale shows the range with red and blue color representing up- and down-regulation respectively. AGI, *A. thaliana* gene IDs provided by TAIR. Gene IDs and gene names with descriptions are provided in Supplementary File S2.

In order to delineate the drought induced changes in plant defenses, the DEGs under drought stress, pathogen infection and combined DP stress were surveyed through MAPMAN and KAAS. Host plant defenses against pathogen characterized by PAMP triggered immunity (PTI) or effector triggered immunity (ETI) and virulence factors were analyzed (Figure 3A). The comparative transcriptome analysis under individual and DP stress shows that the expression pattern of these defense genes under combined stress could not be delineated from either of the individual stresses. These observations suggest that plants elicit different isoforms of a defense gene under individual and combined stresses and possibly opt different route to combat them (Figure 3B). Our results show that the genes involved in PTI were induced upon DP stress where genes involved in MAP kinase signaling were specifically induced in response to bacterial PAMP, flagellin (FLG) recognition, for example, flagellin-sensing 2 (FLS) receptor (Figure 3B). The FLS mediated signaling was however suppressed in pathogen only infected plants. The defense related genes like *AtFRK1* and *PR* were highly induced in response to DP stress while compared to pathogen infection (Figure 3B). ETI related genes however did not show much variation in response to DP stress over pathogen infection. Genes influenced by coronatine, a bacterial virulence factor showed variation in expression pattern. Genes encoding JAZ protein were specifically influenced by different individual and combined stress treatments. *AtJAZ3* and *AtJAZ10* were up-regulated under DP stress, while these were down-regulated under pathogen infection. Apart from PTI, ETI and virulence factor influenced genes, there are certain plant genes which are direct targets of pathogen effectors and contribute to pathogen virulence. These genes were either up-regulated or uninfluenced under drought stress but were mostly down-regulated under pathogen infection (Figure 3B). Comparison with DP stress indicated up-regulation of these genes (Figure 3B).

### Priming with host pathogen suppressed some plant defense responses during combined stress

Nitric oxide-associated protein 1 (NOA1) is involved in nitric oxide biosynthesis and is implicated in various abiotic stresses and pathogen triggered susceptibility (PTS) (Asai and Yoshioka, 2008). The expression of *AtNOA1* was reduced in PD stress (Supplementary Figure S9A). *AtCYP79B3* gene encoding a tryptophan N-hydroxylase is induced by PAMP (Geng et al., 2012) and is involved in the production of precursor of tryptophan-derived metabolites viz., camalexin and glucosinolates (Hull et al., 2000; Gigolashvili et al., 2007). Expression pattern of this gene was also down-regulated in PD stress (Supplementary Figure S9A). Expression pattern of defense genes including TIR/TIR-NBS-LRR class or defensin gene was down-regulated in PD combined stress (Supplementary Figure S9A). Expression of a gene encoding coronatine target protein JAZ10 was also down-regulated under PD stress (Supplementary Figure S9A). Down-regulation of defense genes in PD transcriptome hints toward suppression of some host defenses upon priming with pathogen in combined stress. Thus, although the transcriptome profiles under DP and PD stress were highly correlated, differential expression of the few genes (like those discussed in this section) could possibly underlie the reason for loss of drought induced tolerance under PD stress.

### Drought induced changes in defense related pathways during combined stress

Our analysis revealed up-regulation of genes involved in proline degradation in response to pathogen stress and up-regulation of *AtP5CS1* and down-regulation of *AtProDH2* under drought stress (Supplementary Figure S9B). The observed trend is in line with the involvement of P5C in pathogen defense responses (Qamar et al., 2015) and accumulation of proline under drought stress (Sharma et al., 2011). However, under combined DP stress we observed up-regulation of both proline biosynthesis and proline degradation. This hints toward the maintenance of proline homeostasis under DP stress which seems to be important while sustaining plant growth under stressed condition (Kavi Kishor and Sreenivasulu, 2014).

Likewise, expression profile of polyamine metabolism genes revealed up-regulation of genes involved in polyamine biosynthesis during individual drought and pathogen stresses, but combined stressed plants exhibited up-regulation of both biosynthesis as well as catabolism related genes (Supplementary Figure S9C). Similar observations were made by Hatmi et al. (2014). This points toward the maintenance of polyamine homeostasis under combined stress in order to keep up with the tradeoff between growth and combined stress responses.

The results on expression profile of proline and polyamine metabolism genes thus indicate that the combined stressed plants are preferentially protected against the adverse effects of drought and pathogen induced damages.

## Discussion

Arabidopsis interaction with Pst DC3000 is well studied (Katagiri et al., 2002; Nobuta and Meyers, 2005; Thilmony et al., 2006). Alteration in plant water relations and the modes of physiological responses to reduce the pathogen multiplication in the intracellular spaces has been demonstrated (Freeman and Beattie, 2009; Beattie, 2011). However, the molecular events involved in its interaction in the presence of drought are not known. Importantly, understanding the changes provoked by plants at the interface of simultaneous drought (Osakabe et al., 2014) and pathogen stress is important to know the “net impact” of combined stresses (Ramegowda and Senthil-Kumar, 2015). In this study we showed that the transcriptome profile of *A. thaliana* differs in response to DP and PD combined stresses. We first illustrated the expression of unique batch of genes to combined stress, apart from common genes' expression that are consistent with either of individual stresses studied here. In the later, importantly, we show that a batch of common genes' expression are “tailored” in response to combined stress and their expression pattern is not seen in either of individual stresses. This indicated that plants likely perceive combined stresses as new form of stress, different from individual stresses. One of the previous studies (Choi et al., 2013) on *Vitis vinifera* interaction with simultaneous drought and *X. fastidiosa*, also showed large number of common genes with differential expression pattern and some genes unique to combined stress, indicating that plant uses best possible existing stress responsive molecular machinery for upcoming new or additional stresses, which is rather an economic strategy.

### DP combined stress imposition reduced *In planta* bacterial multiplication

Our study showed that transcriptome changes in drought stressed plants reduced the subsequent pathogen multiplication in DP (over individual pathogen stress), not in PD plants, indicating the time of occurrence of first stress is important determinant of the combined stress interaction with plants (Supplementary Figures S1B,D). We hypothesized that the reduction in pathogen multiplication could be due to change in water relation status of plant physiologically not supporting the pathogen or/and priming for robust defense. Freeman and Beattie (2009) in support of former notion, indicated that localized cessation of water availability in the pathogen infected plants by restricted vascular flow coupled with stomatal loss can reduce bacterial number. Although this was in respect of Pst DC3000 expressing avrRpm1 that provoke gene-for-gene mediated defense responses, the early induction of the localized water limitation in infected tissues could be attributed to basal and/or PAMP-mediated responses. Nevertheless, in our study delivery of pathogen in water by syringe inoculation and only moderate reduction in RWC values of drought stressed plants at the time of pathogen inoculation suggest that water limitation could not be major reason for reduced number of pathogens at 24 hpt (Supplementary Figure S1C). Even the moderate increase in membrane leakage (Supplementary Figure S1E) that could have released more nutrients from the cell in to the apoplast did not facilitate increased multiplication, indicating the possibility of robust defense in the drought stressed plants (DP). Hence, we attribute the later notion, contribution by cell mounted defense, for the reduction in bacterial number as observed in plants responding to drought stress (Osakabe et al., 2014) in our study. In order to explore the details of plant-pathogen interaction under DP, we divided these plant defense responses into three categories namely, non-specific inducible chemical and structural changes, PTI and ETI-mediated changes. Pathogen counter defenses, including virulence factors and ETS are also considered.

### Differential induction of robust defenses likely lead to reduced bacterial number in DP stressed plants

Under DP stress, the plant's unique responses consisted of genes involved in diterpenoid biosynthesis, ABC transporter and inositol phosphate metabolism pathways that contribute to inducible immune responses like callose deposition, cell wall thickening, phenyl propanoids and other secondary metabolite production. Unique genes expressed under combined stresses were specifically enriched with GO biological process “growth.” GO term “response to stress” was prevalent in both unique as well as common genes. These results implies an impact on growth processes while negotiating with the basal stress responses, under combined interaction of two different stressors. Furthermore, expression pattern of stress related genes under DP stress was consistent with drought only gene expression data which indicate that such defense responses are primed in the DP interaction.

Apoplast located pathogen is known to inject effector proteins through type-3 secretion system (Katagiri et al., 2002) and secrete virulence factors (Bender et al., 1999), like coronatine into plant cell to favor its multiplication. Coronatine (COR), JA mimic produced by Pst DC3000 is known to promote this pathogen multiplication and chlorosis by suppressing the SA-mediated defense pathways (Melotto et al., 2008). It is likely that DP combined stressed plants did not suppress SA-pathway as suggested by expression of SA signaling related genes (Supplementary Figure S10). Further, JA related gene expression was high in DP (Supplementary Figure S10). These two evidences can be used to speculate that either the DP plants did not facilitate COR synthesis in pathogen (or its delivery in the plants) or the COR could not facilitate suppression of SA pathway genes which could have been overwhelmed by the SA-JA-ABA hormonal signaling primed by drought stress. Since, the virulence of Pst DC3000 is independent of COR, or at the least only has quantitative effect, we looked at the relevance of effector proteins—their cognate plant receptor genes through DP transcriptome.

Contrary to DP observation, HopAM1 effector had been shown to increase the virulence of an otherwise weak pathogen under drought stress in Wassilewskija (Ws-0) accession of *A. thaliana* (Goel et al., 2008). Our DP microarray profile showed only marginal manipulation of ABA responses (Supplementary Figure S10) that are reported in that study that likely lead to suppression of defense responses in Ws plants. Based on our data, this can be explained by following two ways. First, the Ws-0 is known to have endogenously high and high drought induced ABA levels (North et al., 2007), that suppress several defense responses (Anderson et al., 2004; Fan et al., 2009), compared to Col-0 plants. Second, unlike weak Pma M6CΔE (*P. syringae* pv. maculicola) pathogen strains used in that study, HopAM1 is disposable in Pst DC3000 and virulence was not changed in HopAM1 mutant pathogens. Hence, HopAM1 less likely played a role in combined stress in Col-0. Nevertheless, our observations does not dispel the notion that the diverse effectors produced by Pst DC3000 could exploit the host machinery under drought and favor high infection of a weak pathogen. It is logical to infer that less virulent pathogens could have increased pathogenesis (Kazan and Lyons, 2014), especially in PD like combined stresses. Contrary to the DP, high bacterial multiplication in the PD combined stressed plants indicate that the established pathogen infection before drought stress possibly suppress the plant defense responses.

Further, one of the study on *Verticillium* interaction with *A. thaliana* showed that this pathogen infection enhances drought tolerance (Reusche et al., 2012). Both in DP and PD, we did not find physiological evidences to suggest alteration in the drought specific effects. However, we have observed several changes in the genes attributed to be involved in drought tolerance under stress combination. At this point, we do not have evidences to comment on its impact on either the “net combined stress effect” or the drought-like effect in the combined stressed plants.

## Conclusions

In this manuscript we not only profiled the entire transcriptome changes in combined stressed plants and dissected the unique and shared responses, but also provided brief explanation for the reduced pathogen multiplication, as observed in other plant species, based on the changes in gene regulation. It is likely that both the priming of basal defenses and modulation due to interaction of drought specific and pathogen derived responses in DP plants contributed for this reduction. We also infer that the timing of drought stress decides the outcome of plant interaction with the pathogen infection. Further systematic functional validation studies are needed to elucidate the specific genes involved in the response of DP and PD combined stressed plants.

## Author contributions

MS conceived the idea. MS and AG designed the study. AG performed the experiments. AG analyzed the data with the input from AS. MS and AG wrote the manuscript. AS, AG, and MS critically read the manuscript.

### Conflict of interest statement

The authors declare that the research was conducted in the absence of any commercial or financial relationships that could be construed as a potential conflict of interest.
