# Supplementary material for: Global Transcriptional Analysis Reveals Unique and Shared Responses in Arabidopsis thaliana Exposed to Combined Drought and Pathogen Stress
Source: Front Plant Sci. 2016 May 24;7:686. doi: 10.3389/fpls.2016.00686 (PMC4878317; doi:10.3389/fpls.2016.00686)
Supplement: Supplementary file 1 [file Table1.DOC]

**Supplementary table 1.** Summary of shortlisted genes and primer sequences used in RT-qPCR based validation of microarray data.

| **Gene** | **Oligo name** | **Oligo sequence (5' to 3')** | **Length (bases)** | **Product length (bp)** |
| --- | --- | --- | --- | --- |
| AT3G03341 | UK1-FP | CAAACATTTCATCGGCTTGA | 20 | 107 |
|  | UK1-RP | ACGTCGGTCCTACCACTTTG | 20 |  |
| AT5G01520 | C3HC4-FP | ATGGAGGGATCACCGATGTA | 20 | 103 |
|  | C3HC4-RP | TCGATCTCCGACATCTTTCC | 20 |  |
| AT2G33580 | LYSM-FP | CTTCTTGCCGCTCATACCTC | 20 | 117 |
|  | LYSM-RP | TGGGGAGGTTGTTGATTGAT | 20 |  |
| AT1G07900 | LOBD-FP | TGGAGCCAGCAACATCATTA | 20 | 126 |
|  | LOBD-RP | GCACCTGCACATCCATACAC | 20 |  |
| AT2G18660 | PNPA-FP | GTGACGCTTTTCGGGTTATC | 20 | 195 |
|  | PNPA-RP | CGAATCGAAGCGGATCTAAC | 20 |  |
| AT4G22960 | UK2-FP | GGCCTTGGCATTATCTGAAA | 20 | 148 |
|  | UK2-RP | GGTGGTAGCACAACCAGCTT | 20 |  |
| AT3G25020 | RLP42-FP | GCAACCTAAATCGTCCTTCG | 20 | 166 |
|  | RLP42-RP | CCCATACCCTATTGCCACTG | 20 |  |
| AT1G21460 | SWEET1-FP | CACAGGAGCAGTGATCGAAA | 20 | 116 |
|  | SWEET1-RP | CTGTTGCGAATACAGCCAAA | 20 |  |
| AT5G55460 | NHX3-FP | AACTCCTCCAAGCCGCTAAT | 20 | 197 |
|  | NHX3-RP | TTGCAGGACAAGTTCGAGTG | 20 |  |
| AT3G15350.1 | CORE2.1-FP | GGAACAGAGAGACACACACAAGA | 23 |  |
|  | CORE2.1-RP | TCAAGAACACGGGACACTCC | 20 |  |
| AT5G42570 | BCRA31-FP | CGAACAATGATCCACCTCCT | 20 | 140 |
|  | BCRA31-RP | CGGTGGTTCCTATGGTTTTG | 20 |  |
| AT1G17940 | BRO1-FP | TTCTGTGAAAAATGGGATGCT | 21 | 85 |
|  | BRO1-RP | GAAACCTCACCGATGCTTGT | 20 |  |
| AT4G04220 | RLP46-FP | TCCTGGATATGCGTTTGTGA | 20 | 149 |
|  | RLP46-RP | TCAACGTACCGCCAATAACA | 20 |  |
| AT4G11910 | SG2-FP | TCCCGGAGTACAACAAGGTC | 20 | 127 |
|  | SG2-RP | TTGCACTCATCAGGACAAGG | 20 |  |
| AT3G14590 | NTMC.1-FP | CTTGAATCGATCACACGCGG | 20 | 73 |
|  | NTMC.1-RP | AGAGAAGGAACAACACACCAGG | 22 |  |
| AT1G53887 | RAP2-FP | CGGCGATTCTTGAGGGTGTA | 20 | 178 |
|  | RAP2-RP | ACCATCGTACTCGCTTCATCT | 21 |  |
| AT1G53430 | LRR1-FP | TCGACCGAGAAAGTTGTGTAT | 21 | 99 |
|  | LRR1-RP | TGTTTGAACTTCATCTTCAGGCAA | 24 |  |
| AT4G01540 | NTM1-FP | TAGCAGCGATAGTGGCAGTG | 20 | 119 |
|  | NTM1-RP | CTGTGCTTGAGCAACTTGACA | 21 |  |
| AT1G33480 | ATL58-FP | GATGGCCATGAGCAGCAATG | 20 | 159 |
|  | ATL58-RP | AGCAGCAAGCTTTACGGGAT | 20 |  |
| AT5G23210 | SCPL1-FP | TGGTTACCACCCAGTGAGACT | 21 | 115 |
|  | SCPL1-RP | TCGAATTTGTGGAAATTGTGAA | 22 |  |
| AT3G18780 | *AtACTIN2*-F | ATTCTTGCTTCCCTCAGCAC | 20 | 128 |
|  | *AtACTIN2*-R | CCCCAGCTTTTTAAGCCTTT | 20 |  |
