# Supplementary material for: Global Transcriptional Analysis Reveals Unique and Shared Responses in Arabidopsis thaliana Exposed to Combined Drought and Pathogen Stress
Source: Front Plant Sci. 2016 May 24;7:686. doi: 10.3389/fpls.2016.00686 (PMC4878317; doi:10.3389/fpls.2016.00686)
Supplement: Supplementary file 2 [file Table2.DOCX]

**Supplementary table 2.** Relative enrichment of differentially expressed genes in combined stressed plants (DP) with the individual stress treatment.

| **GO Term** | **Relative enrichment** | |
| --- | --- | --- |
|  | **Unique** | **shared** |
| immune system process | 0.35 | 0.11 |
| metabolic process | 0.75 | 0.77 |
| cellular process | 0.72 | 0.70 |
| signaling | 0.60 | 0.27 |
| multicellular organismal process | 0.74 | 0.00 |
| developmental process | 0.73 | 0.59 |
| growth | 0.61 | 0.00 |
| single-organism process | 0.67 | 0.60 |
| response to stress | 0.58 | 0.43 |
| localization | 0.61 | 0.40 |
| multi-organism process | 0.52 | 0.26 |
| biological regulation | 0.74 | 0.51 |
| cellular component organization or biogenesis | 0.62 | 0.41 |
