# Supplementary material for: Global Transcriptional Analysis Reveals Unique and Shared Responses in Arabidopsis thaliana Exposed to Combined Drought and Pathogen Stress
Source: Front Plant Sci. 2016 May 24;7:686. doi: 10.3389/fpls.2016.00686 (PMC4878317; doi:10.3389/fpls.2016.00686)
Supplement: Supplementary file 3 [file Table3.DOCX]

**Supplementary table 3.** Relative enrichment of differentially expressed genes in combined stressed plants (PD) with the individual stress treatment.

| GO Term | Relative enrichment | |
| --- | --- | --- |
|  | Unique | Common |
| immune system process | 0.47 | 0.15 |
| metabolic process | 0.77 | 0.80 |
| cellular process | 0.75 | 0.75 |
| reproductive process | 0.70 | 0.00 |
| signaling | 0.62 | 0.31 |
| multicellular organismal process | 0.64 | 0.63 |
| developmental process | 0.69 | 0.59 |
| growth | 0.60 | 0.00 |
| single-organism process | 0.74 | 0.64 |
| rhythmic process | 0.28 | 0.00 |
| response to stimulus | 0.64 | 0.47 |
| localization | 0.66 | 0.42 |
| multi-organism process | 0.66 | 0.33 |
| biological regulation | 0.74 | 0.57 |
| cellular component organization or biogenesis | 0.61 | 0.49 |
