# Supplementary material for: Global Transcriptional Analysis Reveals Unique and Shared Responses in Arabidopsis thaliana Exposed to Combined Drought and Pathogen Stress
Source: Front Plant Sci. 2016 May 24;7:686. doi: 10.3389/fpls.2016.00686 (PMC4878317; doi:10.3389/fpls.2016.00686)
Supplement: Supplementary file 4 [file Table4.DOCX]

**Supplementary table 4.** List of pathways mapped uniquely to the DEGs under combined DP stress.

| **Pathway** | **Genes involved** | **Fold change** |
| --- | --- | --- |
| Inositol phosphate metabolism | AT4G39800 (myo-inositol-1-phosphatesynthase1) | -2.427 |
|  | AT3G55940 (phosphoinositide phospholipaseC7) | 2.100 |
| Thiamine metabolism | AT4G15560 (DXS) | -2.272 |
| Folate biosynthesis | AT1G11860 (aminomethyl transferase) | -2.576 |
| Diterpenoid biosynthesis | AT1G61120 (TPS04) | 2.299 |
| ABC transporter | AT3G17820 (glutamine synthetase cytosolic isozyme1-3) | 2.610 |
