# Supplementary material for: Global Transcriptional Analysis Reveals Unique and Shared Responses in Arabidopsis thaliana Exposed to Combined Drought and Pathogen Stress
Source: Front Plant Sci. 2016 May 24;7:686. doi: 10.3389/fpls.2016.00686 (PMC4878317; doi:10.3389/fpls.2016.00686)
Supplement: Supplementary file 5 [file Table5.DOCX]

**Supplementary table 5.** List of pathways mapped uniquely to the DEGs under combined DP stress.

| S. No. | Pathway | Gene | Fold change |
| --- | --- | --- | --- |
|  | Linoleic acid metabolism | AT1G17420 (LOX3) | -2.259 |
|  | Ribosome biogenesis | AT5G27120 ( putative SAR DNA-binding protein) | -2.081 |
|  | Phenylalanine, tyrosine and tryptophan | AT5G53970 (Tyrosine aminotransferase) | 4.267 |
|  | Glycan degradation | AT2G28100 (alpha-L-fucosidase1) | -2.086 |
|  | Nicotinate and nicotinamide | AT4G36940 (nicotinate phosphoribosyl transferase1) | -2.140 |
|  | Monoterpenoid biosynthesis | AT3G61220 [(+)-neomenthol dehydrogenase] | 2.072 |
|  | Limonene and pinene degradation | AT3G26170 (cytochromeP45071B19) | 2.310 |
|  |  | AT3G26280 (cytochromeP45071B4) | 2.243 |
|  | Stilbenoid, diarylheptanoid and gingerol biosynthesis | AT3G26170 (cytochromeP45071B19) | 2.310 |
|  |  | AT3G26280 (cytochromeP45071B4) | 2.243 |
|  |  | AT1G24764 (microtubule-associated protein70-2) | -2.129 |
|  | Isoquinoline alkaloid biosynthesis | AT5G53970 (tyrosine aminotransferase ) | 4.267 |
|  | lysosome | AT4G01610 (putative cathepsinB-like cysteine protease) | 2.151 |
|  | DNA replication | AT2G25100 (ribonuclease H2 subunitA) | -2.053 |
|  | Nucleotide excision repair | AT5G58760 (DNA damage-binding protein2) | 2.951 |
|  | RNA pol, splicesome | ATCG00170 (RNA polymerase beta''chain) | -3.332 |
|  |  | ATCG00180 (RNA polymerase beta'chain) | -2.510 |
|  |  | ATCG00190 (RNA polymerase beta'chain) | -2.743 |
|  |  | AT4G30330 (small nuclear ribonucleoproteinE) | -2.107 |
|  |  | AT4G24280 (chloroplast heat shock protein70-1) | -2.741 |
|  | Aminoacyl TRNA biosynthesis | AT5G22800 (probablealanine—tRNA ligase) | -2.289 |
|  |  | AT5G16715 (ATP binding/valine-tRNA ligase/aminoacyl-tRNA ligase) | -2.775 |
|  |  | AT5G49030 (disease resistance-responsive (dirigent-like protein familyprotein) | -2.441 |
|  |  | AT2G31170 (cysteinyl-tRNA synthetase) | -2.193 |
|  | RNA transport and protein export | AT4G31120 (protein arginineN-methyltransferase5) | -2.328 |
|  |  | AT5G54940 (translation initiation factor SUI1familyprotein) | 3.079 |
|  |  | AT2G18710 (preprotein translocasesubunitsecY) | -2.674 |
|  |  | AT4G30600 (signal recognition particle receptor subunit alpha) | 2.206 |
|  |  | AT2G01110 (Sec-independentproteintranslocaseproteinTATC) | -2.074 |
|  | mRNA surveillance pathway | AT5G43620 (Pre-mRNAcleavagecomplexII) | 2.398 |
|  | Linoleic acid metabolism | AT1G17420 (LOX3) | -2.259 |
|  | Ribosome biogenesis | AT5G27120 ( putative SAR DNA-binding protein) | -2.081 |
|  | Phenylalanine,tyrosine and tryptophan | AT5G53970 (Tyrosine aminotransferase) | 4.267 |
|  | Glycan degradation | AT2G28100 (alpha-L-fucosidase1) | -2.086 |
|  | Nicotinate and nicotinamide | AT4G36940 (nicotinatephosphoribosyltransferase1) | -2.140 |
|  | Monoterpenoid biosynthesis | AT3G61220 [(+)-neomentholdehydrogenase] | 2.072 |
|  | Limonene and pinene degradation | AT3G26170 (cytochromeP45071B19) | 2.310 |
|  |  | AT3G26280 (cytochromeP45071B4) | 2.243 |
|  | Stilbenoid, diarylheptanoid and gingerol biosynthesis | AT3G26170 (cytochromeP45071B19) | 2.310 |
|  |  | AT3G26280 (cytochromeP45071B4) | 2.243 |
|  |  | AT1G24764 (microtubule-associated protein70-2) | -2.129 |
|  | Isoquinoline alkaloid biosynthesis | AT5G53970 (tyrosine aminotransferase ) | 4.267 |
|  | lysosome | AT4G01610 (putative cathepsin B-like cysteine protease) | 2.151 |
|  | DNA replication | AT2G25100 (ribonuclease H2 subunitA) | -2.053 |
|  | Nucleotide excision repair | AT5G58760 (DNA damage-binding protein2) | 2.951 |
|  | RNA pol, splicesome | ATCG00170 (RNA polymerase beta''chain) | -3.332 |
|  |  | ATCG00180 (RNA polymerase beta'chain) | -2.510 |
|  |  | ATCG00190 (RNA polymerase beta'chain) | -2.743 |
|  |  | AT4G30330 (small nuclear ribonucleoprotein E) | -2.107 |
|  |  | AT4G24280 (chloroplast heatshock protein70-1) | -2.741 |
|  | Aminoacyl TRNA biosynthesis | AT5G22800probablealanine—tRNA ligase | -2.289 |
|  |  | AT5G16715 (ATP binding/valine-tRNAligase/aminoacyl-tRNA ligase) | -2.775 |
|  |  | AT5G49030 (isoleucyl-tRNAsynthetase\|diseaseresistance-responsive(dirigent-likeprotein)familyprotein) | -2.441 |
|  |  | AT2G31170 (cysteinyl-tRNA synthetase) | -2.193 |
|  | RNA transport and protein export | AT4G31120 (proteinarginineN-methyltransferase5) | -2.328 |
|  |  | AT5G54940 (translationinitiationfactorSUI1familyprotein) | 3.079 |
|  |  | AT2G18710 (preproteintranslocasesubunitsecY) | -2.674 |
|  |  | AT4G30600 (signalrecognitionparticlereceptorsubunitalpha) | 2.206 |
|  |  | AT2G01110 (Sec-independentproteintranslocaseproteinTATC) | -2.074 |
|  | mRNA surveillance pathway | AT5G43620 (Pre-mRNAcleavagecomplexII) | 2.398 |
