# Supplementary material for: Global Transcriptional Analysis Reveals Unique and Shared Responses in Arabidopsis thaliana Exposed to Combined Drought and Pathogen Stress
Source: Front Plant Sci. 2016 May 24;7:686. doi: 10.3389/fpls.2016.00686 (PMC4878317; doi:10.3389/fpls.2016.00686)
Supplement: Supplementary file 6 [file Presentation1.PPTX]

## Slide 1
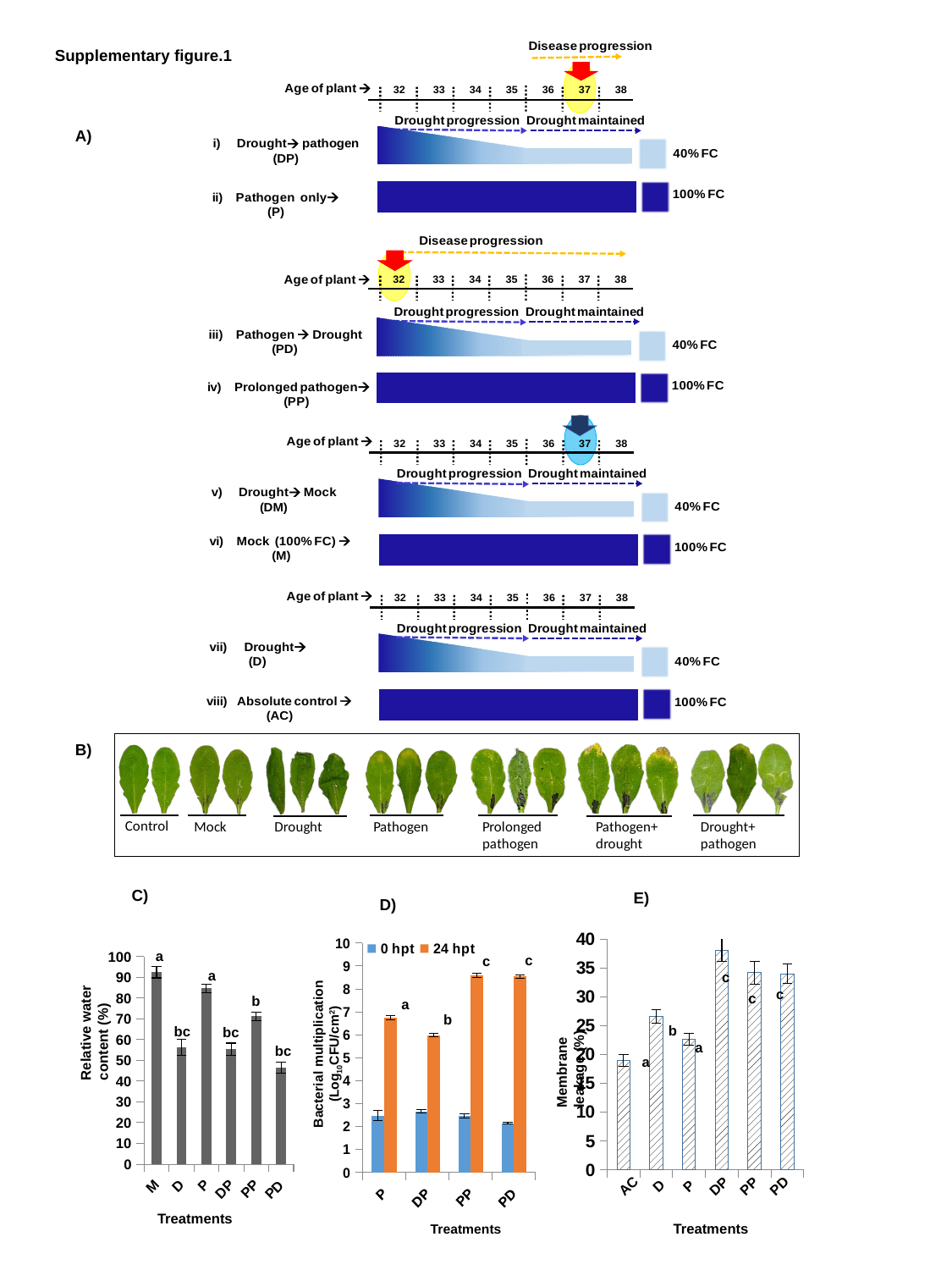

Supplementary figure.1
A)
B)
C)
E)
D)
### Chart
| Category | 0 hpt | 24 hpt |
|---|---|---|
| P | 2.481499878197121 | 6.744026411542571 |
| DP | 2.658576049818999 | 5.9741326064124785 |
| PP | 2.4506525351209567 | 8.589705281361496 |
| PD | 2.148625708805531 | 8.5489895559221 |Bacterial multiplication (Log10CFU/cm2)
Treatments
### Chart
| Category | EC |
|---|---|
| AC | 18.91005900245253 |
| D | 26.588106172244913 |
| P | 22.63203572491973 |
| D+P | 38.081595172504265 |
| PP | 34.194277699400494 |
| P+D | 33.98947293477141 |Membrane leakage (%)
Treatments
AC
D
P
DP
PP
PD
c
c
c
c
b
a
a
Relative water content (%)
Treatments
### Chart
| Category | RWC |
|---|---|
| M | 92.56563333559882 |
| D | 56.14655944674 |
| P | 84.660024328983 |
| DP | 55.308964389356 |
| PP | 71.23378127559384 |
| PD | 46.549810659324294 |a
a
b
bc
bc
bc
c
a
b

## Slide 2
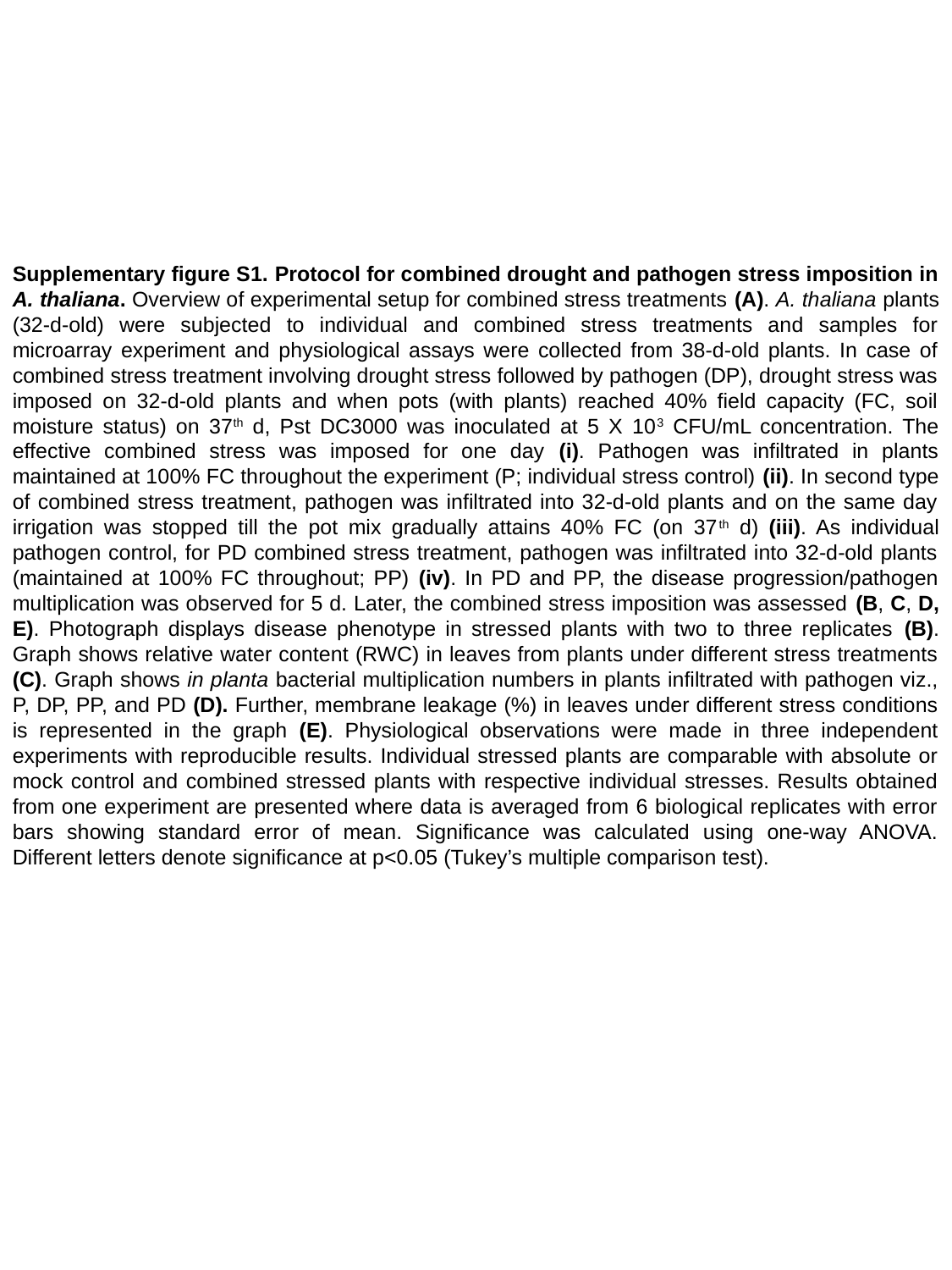

Supplementary figure S1. Protocol for combined drought and pathogen stress imposition in A. thaliana. Overview of experimental setup for combined stress treatments (A). A. thaliana plants (32-d-old) were subjected to individual and combined stress treatments and samples for microarray experiment and physiological assays were collected from 38-d-old plants. In case of combined stress treatment involving drought stress followed by pathogen (DP), drought stress was imposed on 32-d-old plants and when pots (with plants) reached 40% field capacity (FC, soil moisture status) on 37th d, Pst DC3000 was inoculated at 5 X 103 CFU/mL concentration. The effective combined stress was imposed for one day (i). Pathogen was infiltrated in plants maintained at 100% FC throughout the experiment (P; individual stress control) (ii). In second type of combined stress treatment, pathogen was infiltrated into 32-d-old plants and on the same day irrigation was stopped till the pot mix gradually attains 40% FC (on 37th d) (iii). As individual pathogen control, for PD combined stress treatment, pathogen was infiltrated into 32-d-old plants (maintained at 100% FC throughout; PP) (iv). In PD and PP, the disease progression/pathogen multiplication was observed for 5 d. Later, the combined stress imposition was assessed (B, C, D, E). Photograph displays disease phenotype in stressed plants with two to three replicates (B). Graph shows relative water content (RWC) in leaves from plants under different stress treatments (C). Graph shows in planta bacterial multiplication numbers in plants infiltrated with pathogen viz., P, DP, PP, and PD (D). Further, membrane leakage (%) in leaves under different stress conditions is represented in the graph (E). Physiological observations were made in three independent experiments with reproducible results. Individual stressed plants are comparable with absolute or mock control and combined stressed plants with respective individual stresses. Results obtained from one experiment are presented where data is averaged from 6 biological replicates with error bars showing standard error of mean. Significance was calculated using one-way ANOVA. Different letters denote significance at p<0.05 (Tukey’s multiple comparison test).
