# Supplementary material for: Global Transcriptional Analysis Reveals Unique and Shared Responses in Arabidopsis thaliana Exposed to Combined Drought and Pathogen Stress
Source: Front Plant Sci. 2016 May 24;7:686. doi: 10.3389/fpls.2016.00686 (PMC4878317; doi:10.3389/fpls.2016.00686)
Supplement: Supplementary file 7 [file Presentation2.PPTX]

## Slide 1
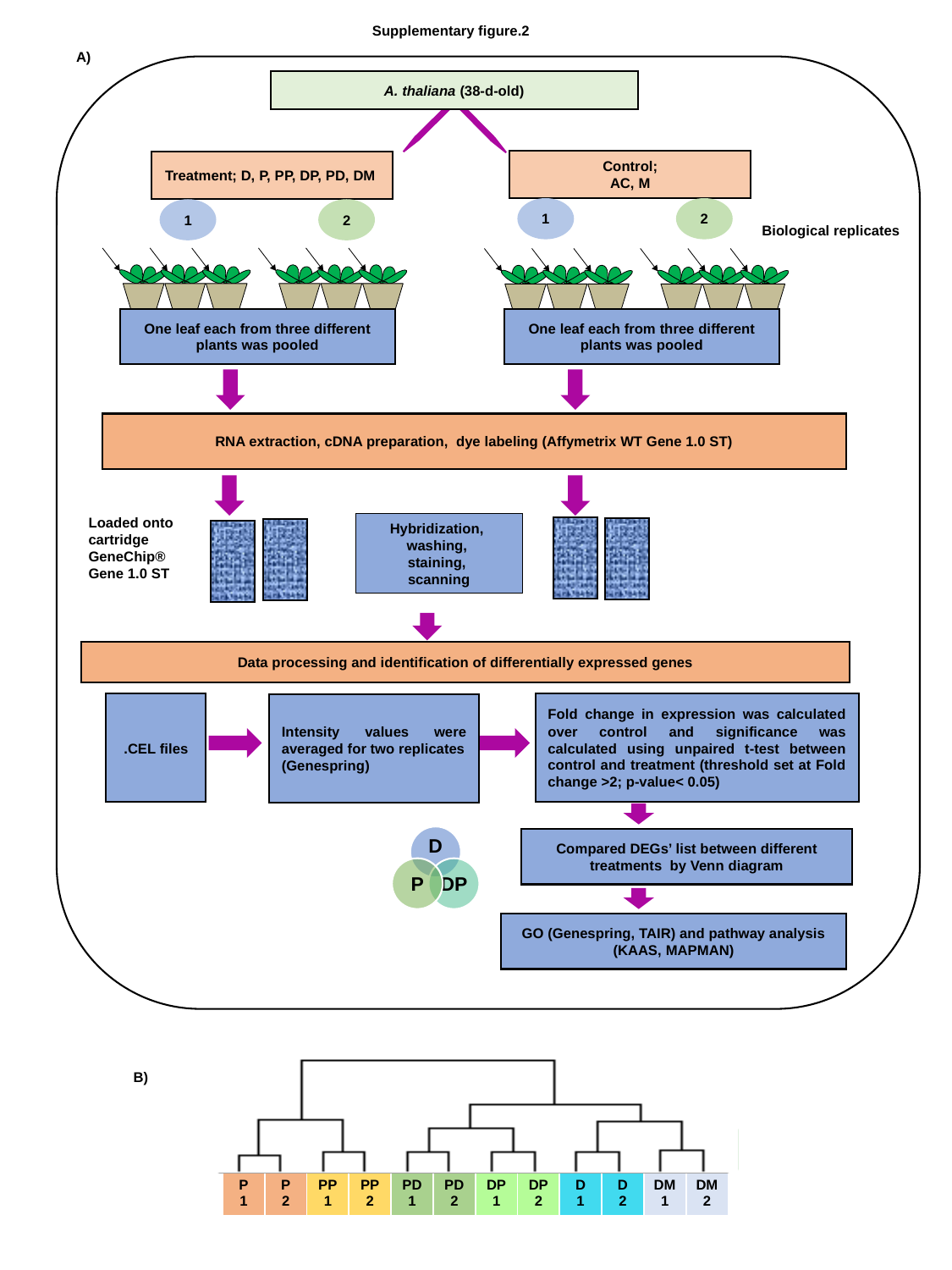

Supplementary figure.2
A)
A. thaliana (38-d-old)
Control;
AC, M
Treatment; D, P, PP, DP, PD, DM
1
2
1
2
Biological replicates
One leaf each from three different plants was pooled
One leaf each from three different plants was pooled
RNA extraction, cDNA preparation, dye labeling (Affymetrix WT Gene 1.0 ST)
Hybridization,
washing,
staining,
scanning
Data processing and identification of differentially expressed genes
.CEL files
Fold change in expression was calculated over control and significance was calculated using unpaired t-test between control and treatment (threshold set at Fold change >2; p-value< 0.05)
Intensity values were averaged for two replicates
(Genespring)
Compared DEGs’ list between different treatments by Venn diagram
GO (Genespring, TAIR) and pathway analysis (KAAS, MAPMAN)
Loaded onto cartridge
GeneChip® Gene 1.0 ST
B)
| P 1 | P 2 | PP 1 | PP 2 | PD 1 | PD 2 | DP 1 | DP 2 | D 1 | D 2 | DM 1 | DM 2 |
| --- | --- | --- | --- | --- | --- | --- | --- | --- | --- | --- | --- |

## Slide 2
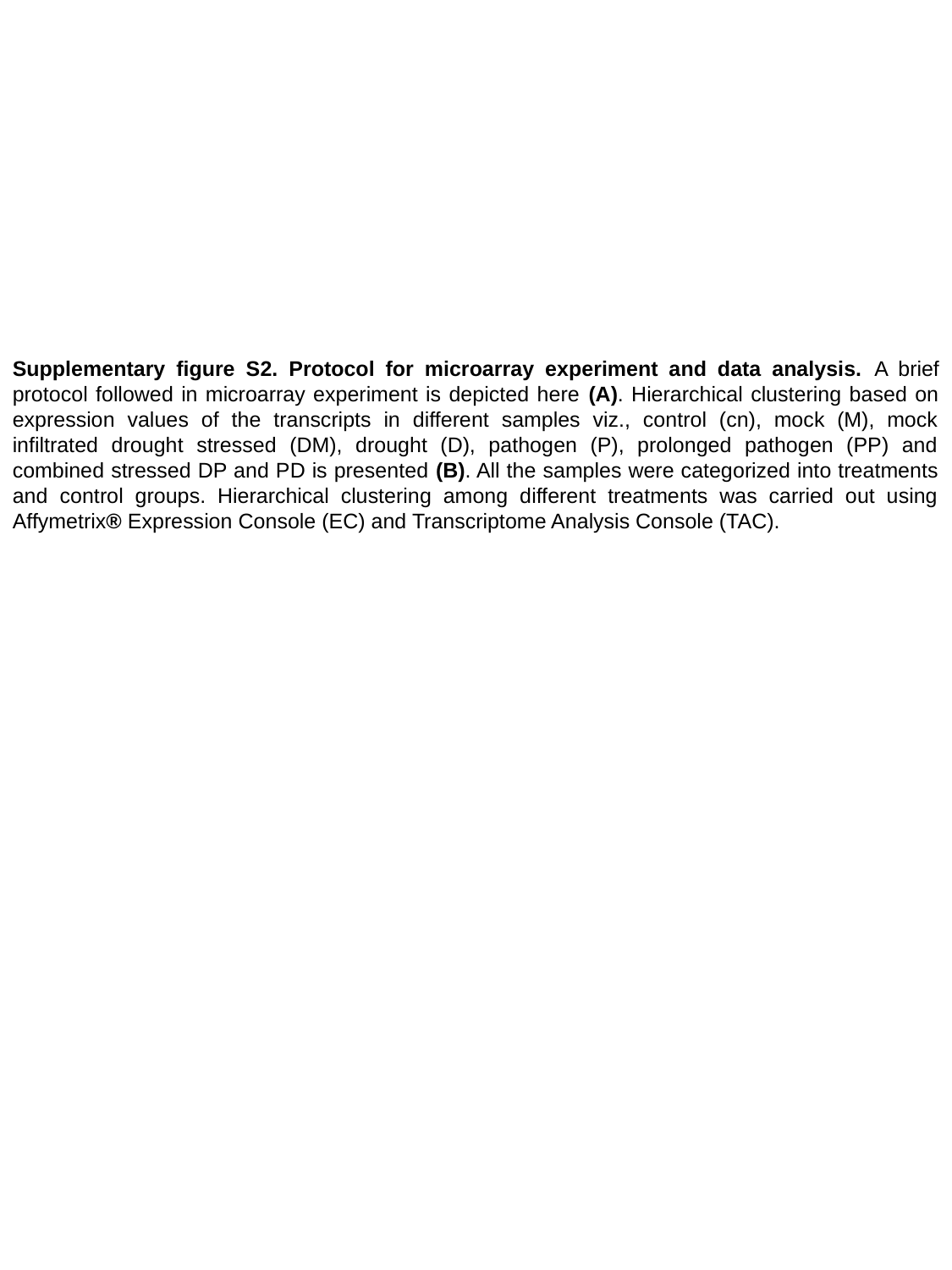

Supplementary figure S2. Protocol for microarray experiment and data analysis. A brief protocol followed in microarray experiment is depicted here (A). Hierarchical clustering based on expression values of the transcripts in different samples viz., control (cn), mock (M), mock infiltrated drought stressed (DM), drought (D), pathogen (P), prolonged pathogen (PP) and combined stressed DP and PD is presented (B). All the samples were categorized into treatments and control groups. Hierarchical clustering among different treatments was carried out using Affymetrix® Expression Console (EC) and Transcriptome Analysis Console (TAC).
