# Supplementary material for: Global Transcriptional Analysis Reveals Unique and Shared Responses in Arabidopsis thaliana Exposed to Combined Drought and Pathogen Stress
Source: Front Plant Sci. 2016 May 24;7:686. doi: 10.3389/fpls.2016.00686 (PMC4878317; doi:10.3389/fpls.2016.00686)
Supplement: Supplementary file 8 [file Presentation3.PPTX]

## Slide 1
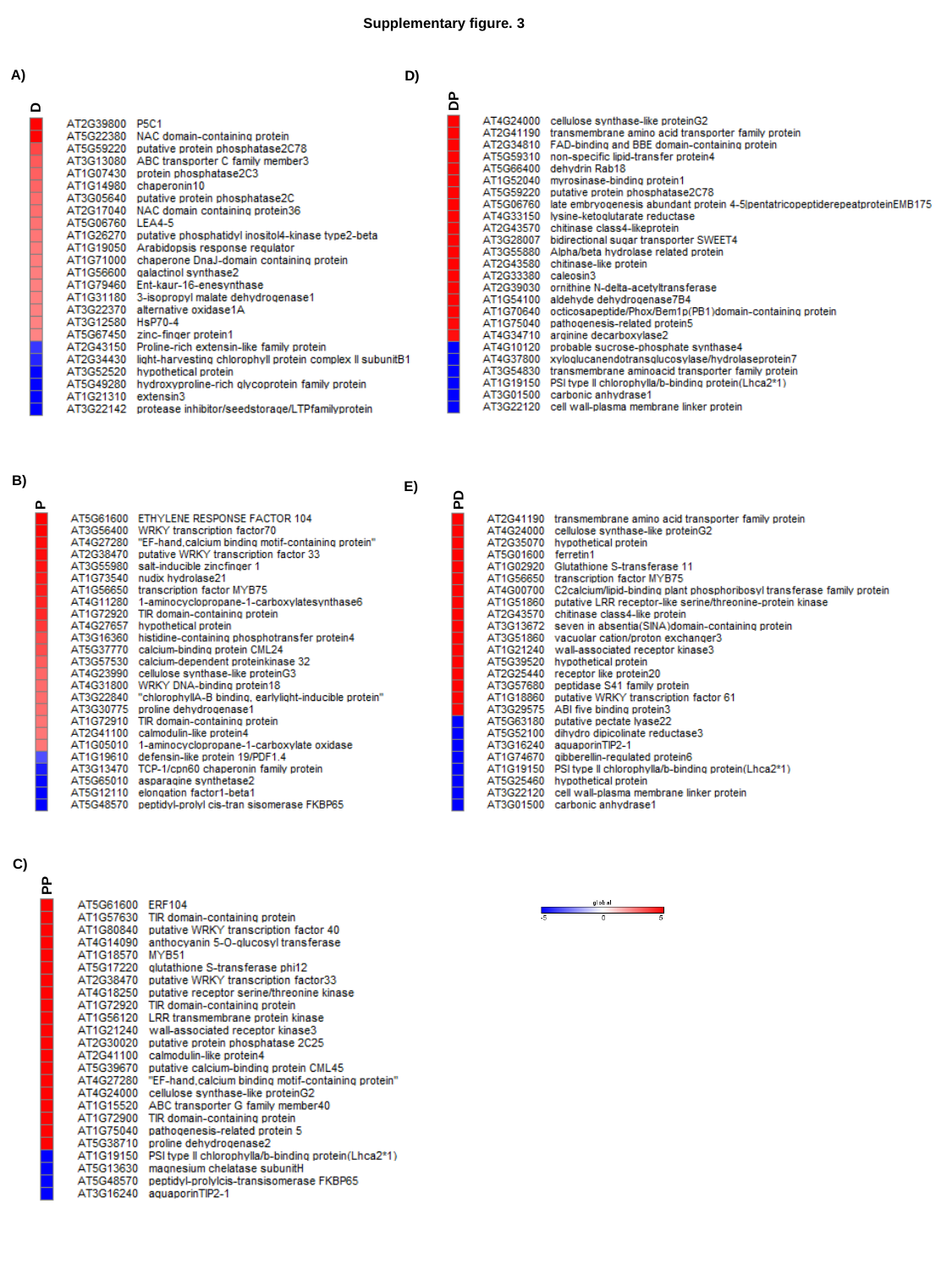

Supplementary figure. 3
A)
D)
DP
D
B)
P
PD
E)
C)
PP

## Slide 2
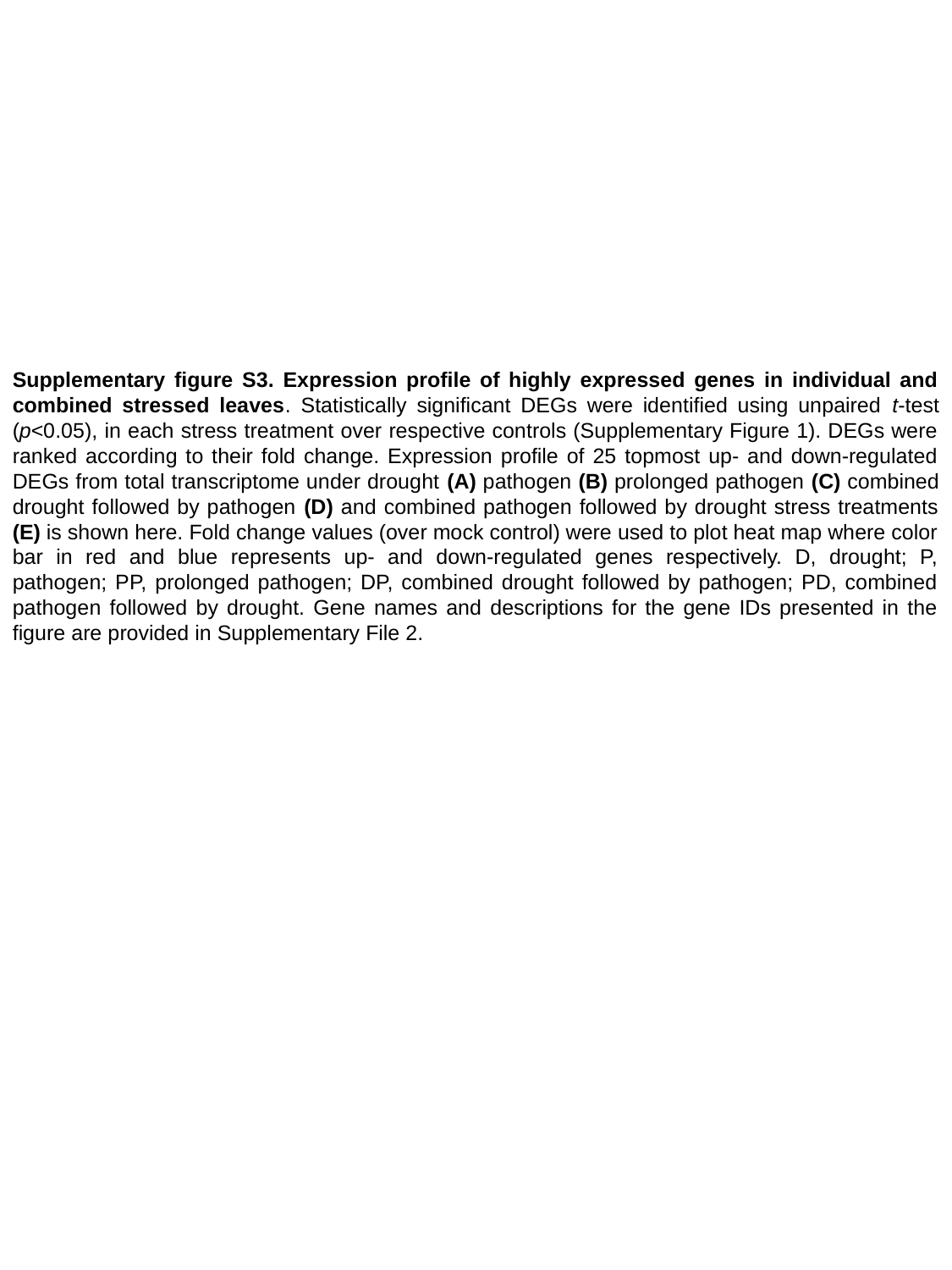

Supplementary figure S3. Expression profile of highly expressed genes in individual and combined stressed leaves. Statistically significant DEGs were identified using unpaired t-test (p<0.05), in each stress treatment over respective controls (Supplementary Figure 1). DEGs were ranked according to their fold change. Expression profile of 25 topmost up- and down-regulated DEGs from total transcriptome under drought (A) pathogen (B) prolonged pathogen (C) combined drought followed by pathogen (D) and combined pathogen followed by drought stress treatments (E) is shown here. Fold change values (over mock control) were used to plot heat map where color bar in red and blue represents up- and down-regulated genes respectively. D, drought; P, pathogen; PP, prolonged pathogen; DP, combined drought followed by pathogen; PD, combined pathogen followed by drought. Gene names and descriptions for the gene IDs presented in the figure are provided in Supplementary File 2.
