# Supplementary material for: Global Transcriptional Analysis Reveals Unique and Shared Responses in Arabidopsis thaliana Exposed to Combined Drought and Pathogen Stress
Source: Front Plant Sci. 2016 May 24;7:686. doi: 10.3389/fpls.2016.00686 (PMC4878317; doi:10.3389/fpls.2016.00686)
Supplement: Supplementary file 9 [file Presentation4.PPTX]

## Slide 1
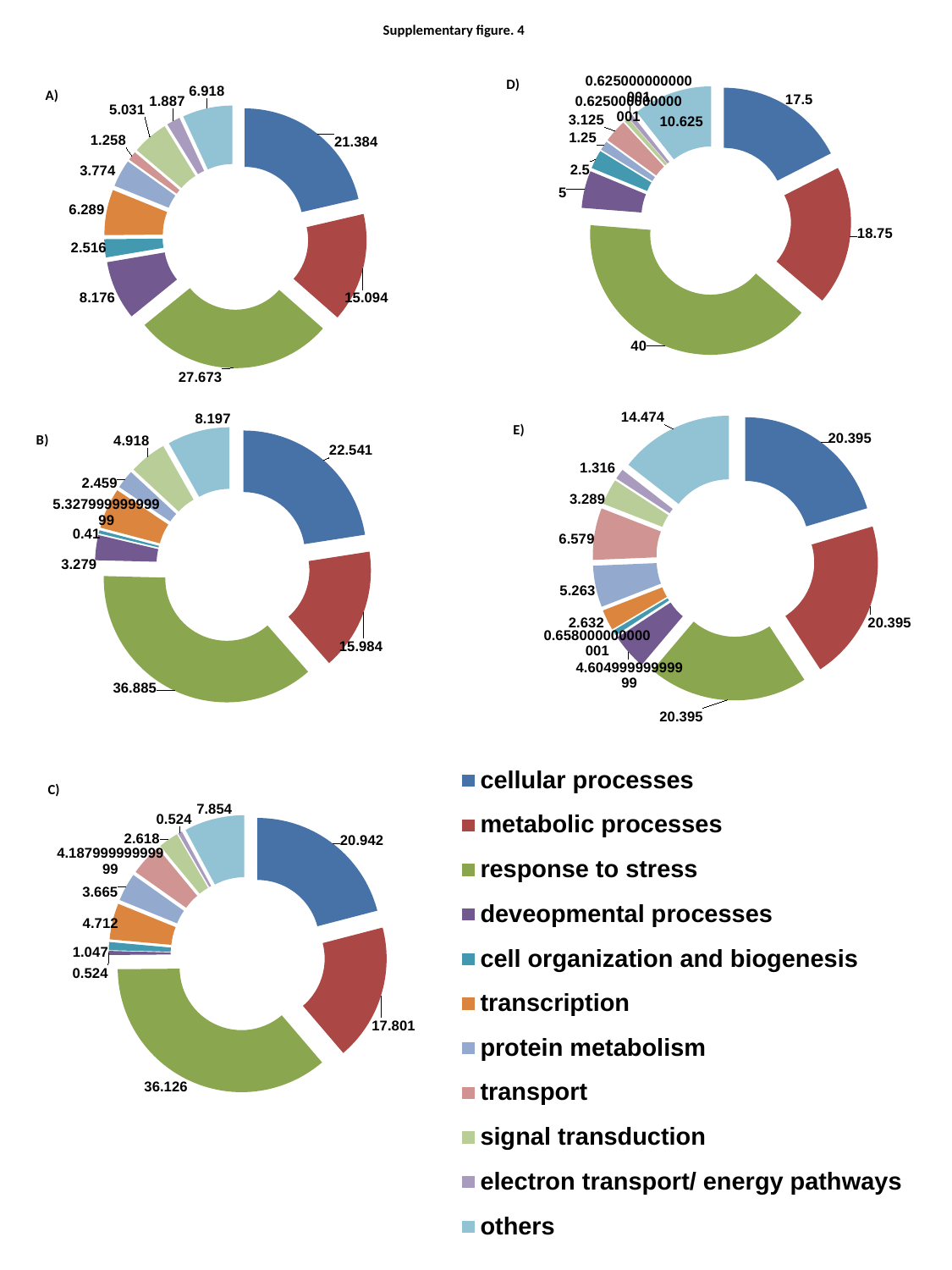

Supplementary figure. 4
### Chart
| Category | DP |
|---|---|
| cellular processes | 17.5 |
| metabolic processes | 18.75 |
| response to stress | 40.0 |
| deveopmental processes | 5.0 |
| cell organization and biogenesis | 2.5 |
| transcription | 0.0 |
| protein metabolism | 1.25 |
| transport | 3.125 |
| signal transduction | 0.625000000000001 |
| electron transport/ energy pathways | 0.625000000000001 |
| others | 10.625 |D)
### Chart
| Category | D ALL |
|---|---|
| cellular processes | 21.384 |
| metabolic processes | 15.094000000000001 |
| response to stress | 27.673000000000005 |
| deveopmental processes | 8.176 |
| cell organization and biogenesis | 2.5159999999999987 |
| transcription | 6.289 |
| protein metabolism | 3.774 |
| transport | 1.258 |
| signal transduction | 5.031 |
| electron transport/ energy pathways | 1.887 |
| others | 6.918 |A)
### Chart
| Category | PD |
|---|---|
| cellular processes | 20.395 |
| metabolic processes | 20.395 |
| response to stress | 20.395000000000003 |
| deveopmental processes | 4.604999999999993 |
| cell organization and biogenesis | 0.6580000000000011 |
| transcription | 2.6319999999999997 |
| protein metabolism | 5.2629999999999955 |
| transport | 6.579 |
| signal transduction | 3.289 |
| electron transport/ energy pathways | 1.316 |
| others | 14.474 |
### Chart
| Category | P |
|---|---|
| cellular processes | 22.541 |
| metabolic processes | 15.984 |
| response to stress | 36.885000000000005 |
| deveopmental processes | 3.279 |
| cell organization and biogenesis | 0.4100000000000003 |
| transcription | 5.327999999999991 |
| protein metabolism | 2.4589999999999987 |
| transport | 0.0 |
| signal transduction | 4.918 |
| electron transport/ energy pathways | 0.0 |
| others | 8.197000000000001 |E)
B)
### Chart
| Category | PP |
|---|---|
| cellular processes | 20.941999999999986 |
| metabolic processes | 17.800999999999988 |
| response to stress | 36.12600000000001 |
| deveopmental processes | 0.524 |
| cell organization and biogenesis | 1.0469999999999982 |
| transcription | 4.712 |
| protein metabolism | 3.665 |
| transport | 4.187999999999993 |
| signal transduction | 2.618 |
| electron transport/ energy pathways | 0.524 |
| others | 7.8539999999999965 |
C)

## Slide 2
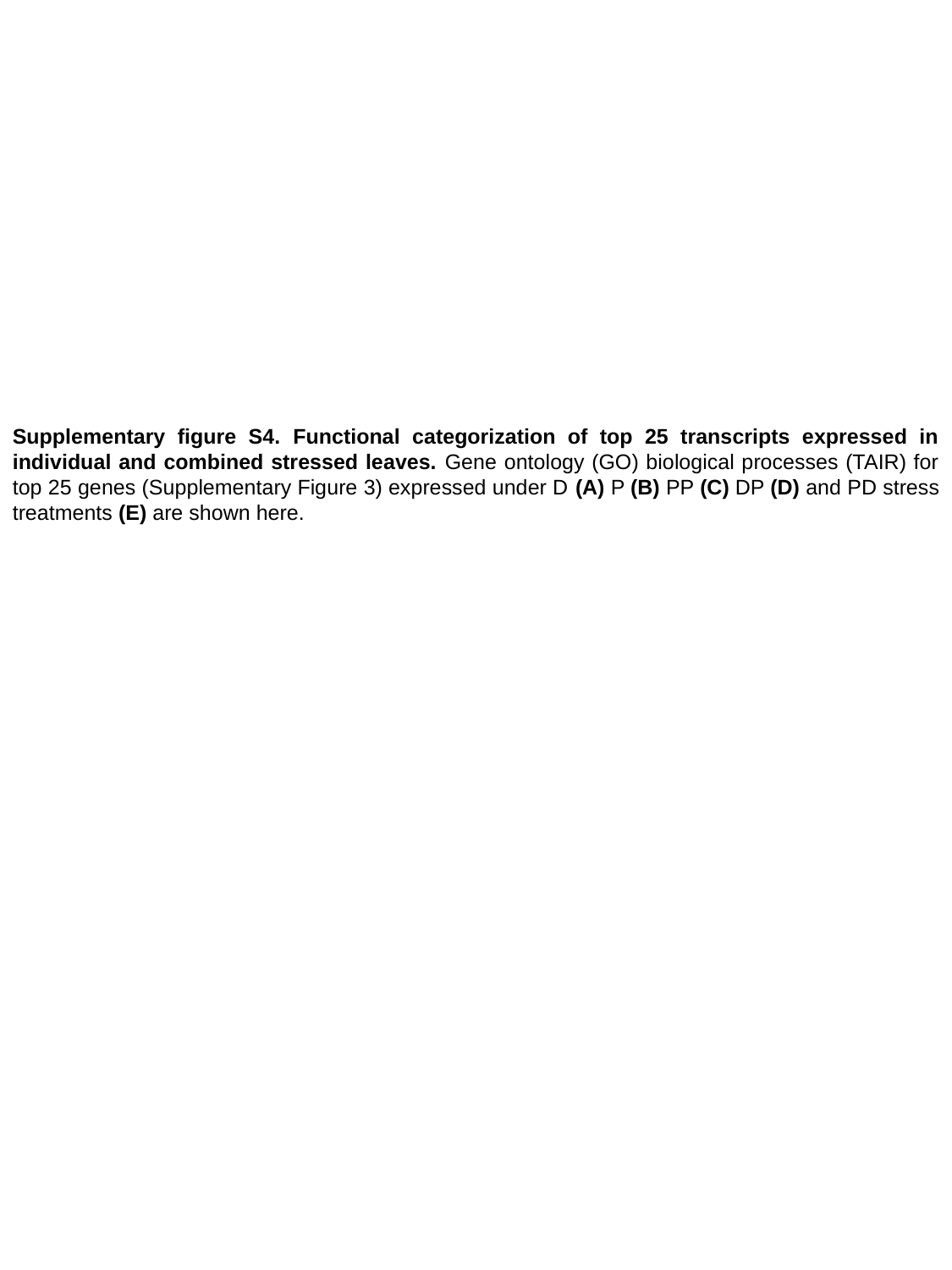

Supplementary figure S4. Functional categorization of top 25 transcripts expressed in individual and combined stressed leaves. Gene ontology (GO) biological processes (TAIR) for top 25 genes (Supplementary Figure 3) expressed under D (A) P (B) PP (C) DP (D) and PD stress treatments (E) are shown here.
