# Supplementary material for: Global Transcriptional Analysis Reveals Unique and Shared Responses in Arabidopsis thaliana Exposed to Combined Drought and Pathogen Stress
Source: Front Plant Sci. 2016 May 24;7:686. doi: 10.3389/fpls.2016.00686 (PMC4878317; doi:10.3389/fpls.2016.00686)
Supplement: Supplementary file 10 [file Presentation5.PPTX]

## Slide 1
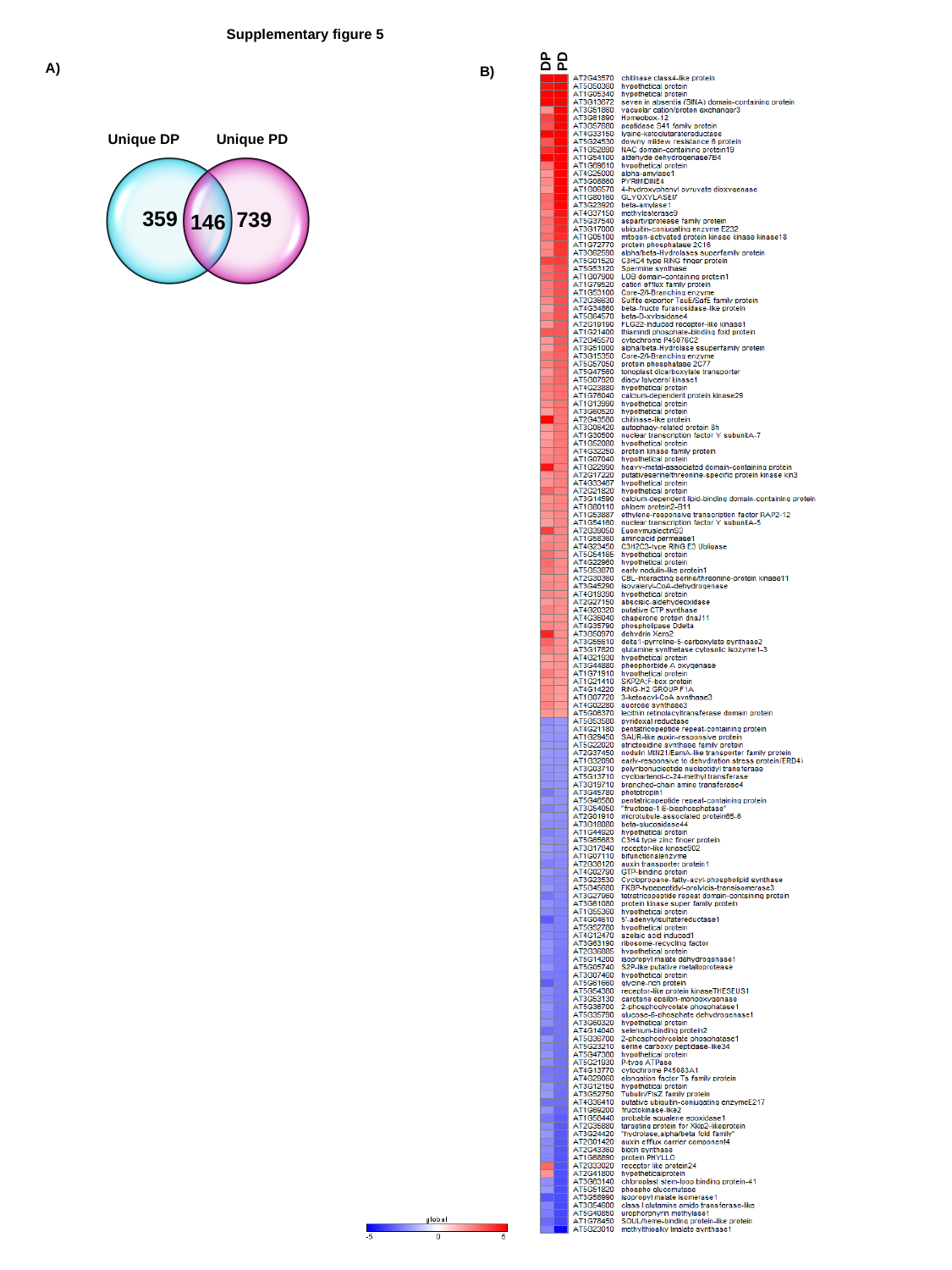

Supplementary figure 5
DP
PD
A)
B)
Unique DP
Unique PD

## Slide 2
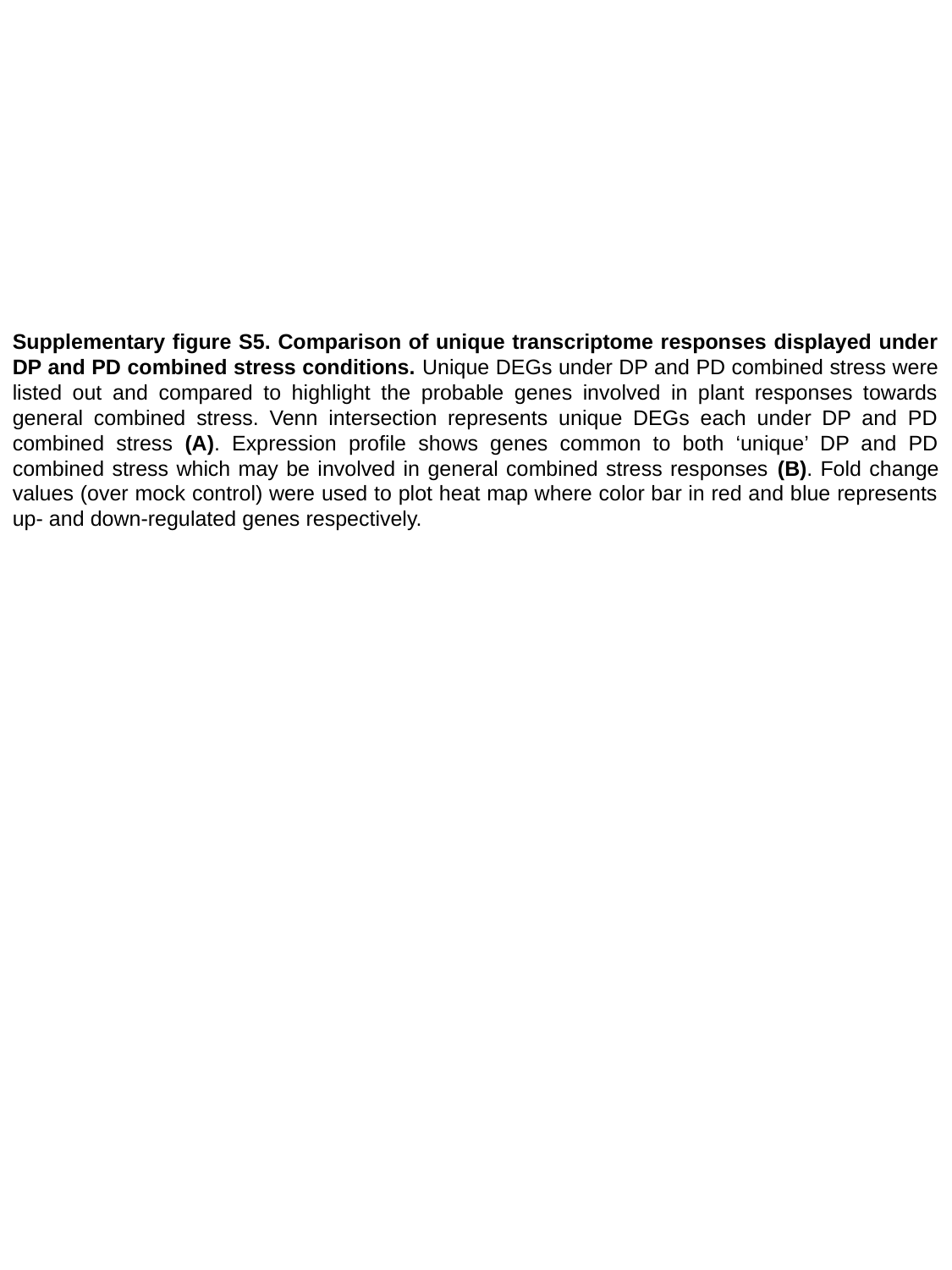

Supplementary figure S5. Comparison of unique transcriptome responses displayed under DP and PD combined stress conditions. Unique DEGs under DP and PD combined stress were listed out and compared to highlight the probable genes involved in plant responses towards general combined stress. Venn intersection represents unique DEGs each under DP and PD combined stress (A). Expression profile shows genes common to both ‘unique’ DP and PD combined stress which may be involved in general combined stress responses (B). Fold change values (over mock control) were used to plot heat map where color bar in red and blue represents up- and down-regulated genes respectively.
