# Supplementary material for: Global Transcriptional Analysis Reveals Unique and Shared Responses in Arabidopsis thaliana Exposed to Combined Drought and Pathogen Stress
Source: Front Plant Sci. 2016 May 24;7:686. doi: 10.3389/fpls.2016.00686 (PMC4878317; doi:10.3389/fpls.2016.00686)
Supplement: Supplementary file 11 [file Presentation6.PPTX]

## Slide 1
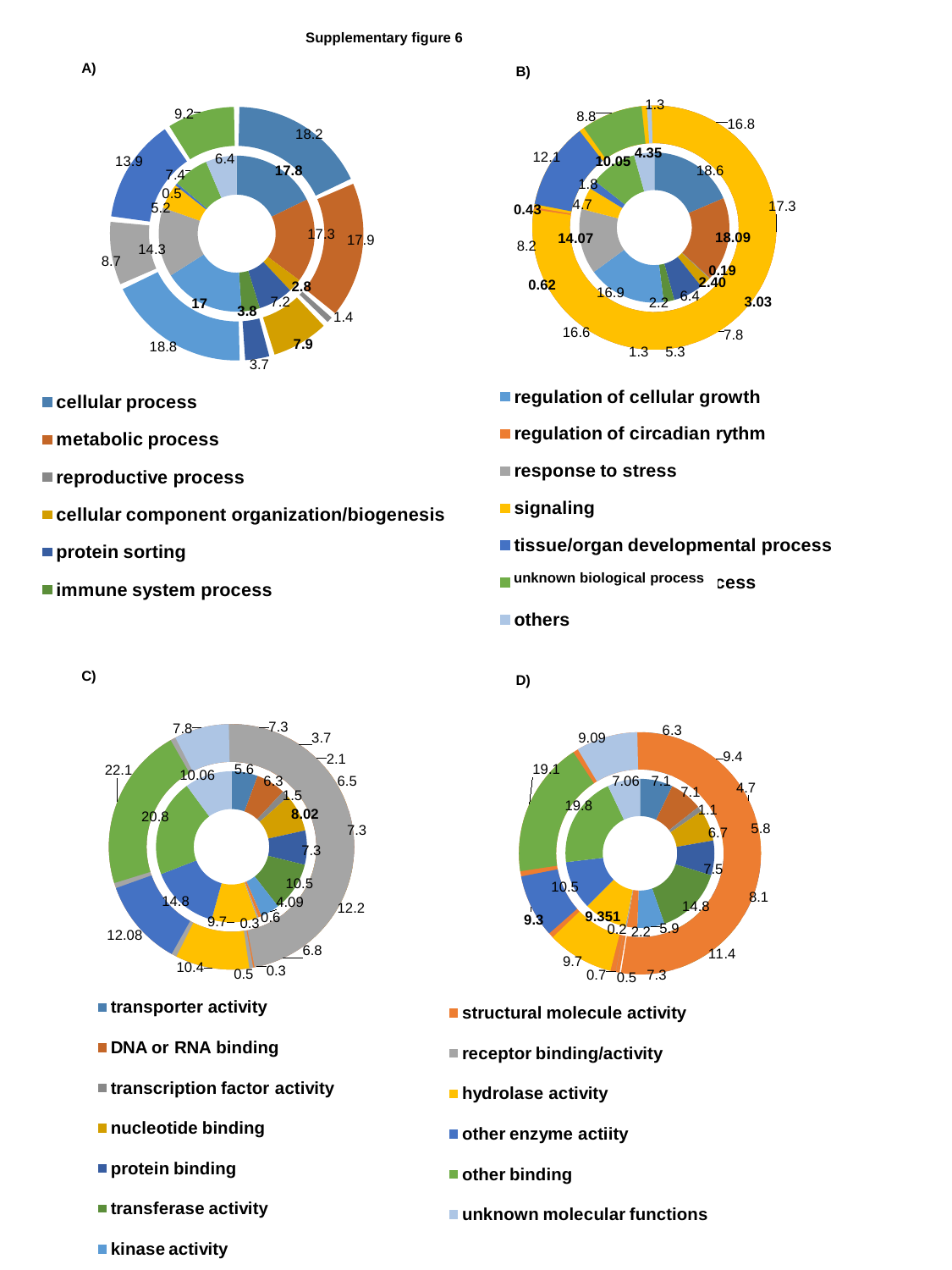

Supplementary figure 6
A)
B)
### Chart
| Category | DP Up | DP Down |
|---|---|---|
| cellular process | 17.81 | 18.22 |
| metabolic process | 17.36 | 17.95 |
| Reproductive process | 0.0 | 1.44 |
| cellular component organization or biogenesis | 2.8 | 7.9 |
| protein sorting | 7.23 | 3.77 |
| immune system process | 3.8 | None |
| regulation of cellular growth | 17.0 | 18.86 |
| regulation of circadian rythm | 0.0 | 0.0 |
| response to stress | 14.38 | 8.71 |
| signaling | 5.24 | 0.0 |
| tissue/organ developmental process | 0.54 | 13.91 |
| unknown biologicalprocess | 7.41 | 9.25 |
| others | 6.42 | 0.0 |
### Chart
| Category | PD Up | PD down |
|---|---|---|
| cellular process | 18.61000000000003 | 16.86 |
| metabolic process | 18.09 | 17.34 |
| reproductive process | 0.19 | 3.03 |
| cellular component organization/biogenesis | 2.4 | 7.8599999999999985 |
| protein sorting | 6.42 | 5.35 |
| immune system process | 2.27 | 1.37 |
| regulation of cellular growth | 16.93 | 16.670000000000005 |
| regulation of circadian rythm | 0.0 | 0.6200000000000012 |
| response to stress | 14.07 | 8.200000000000001 |
| signaling | 4.73 | 0.4300000000000004 |
| tissue/organ developmental process | 1.8800000000000001 | 12.129999999999999 |
| unknown biologicalprocess | 10.050000000000002 | 8.81 |
| others | 4.35 | 1.33 |
unknown biological process
### Chart
| Category | DP DOWN | DP UP |
|---|---|---|
| transporter activity | 5.631 | 7.383 |
| DNA or RNA binding | 6.3139999999999965 | 3.7246 |
| transcription factor activity | 1.536 | 2.181 |
| nucleotide binding | 8.02 | 6.544 |
| protein binding | 7.338 | 7.383 |
| transferase activity | 10.58 | 12.248 |
| kinase activity | 4.069 | 6.879 |
| structural molecule activity | 0.683 | 0.33600000000000074 |
| receptor binding/activity | 0.3410000000000001 | 0.503 |
| hydrolase activity | 9.727 | 10.403 |
| other enzyme actiity | 14.846 | 12.081 |
| other binding | 20.819 | 22.148 |
| unknown molecular functions | 10.068 | 7.8859999999999975 |
### Chart
| Category | PD DOWN | PD UP |
|---|---|---|
| transporter activity | 7.155999999999991 | 6.374 |
| DNA or RNA binding | 7.1569999999999965 | 9.404000000000003 |
| transcription factor activity | 1.145 | 4.702 |
| nucleotide binding | 6.775 | 5.8519999999999985 |
| protein binding | 7.538 | 8.15 |
| transferase activity | 14.885000000000018 | 11.494 |
| kinase activity | 5.916 | 7.314999999999991 |
| structural molecule activity | 2.29 | 0.522 |
| receptor binding/activity | 0.2860000000000003 | 0.7310000000000006 |
| hydrolase activity | 9.351000000000003 | 9.741 |
| other enzyme actiity | 10.592000000000002 | 9.3 |
| other binding | 19.847 | 19.122 |
| unknown molecular functions | 7.0609999999999955 | 9.091000000000001 |
C)
D)

## Slide 2
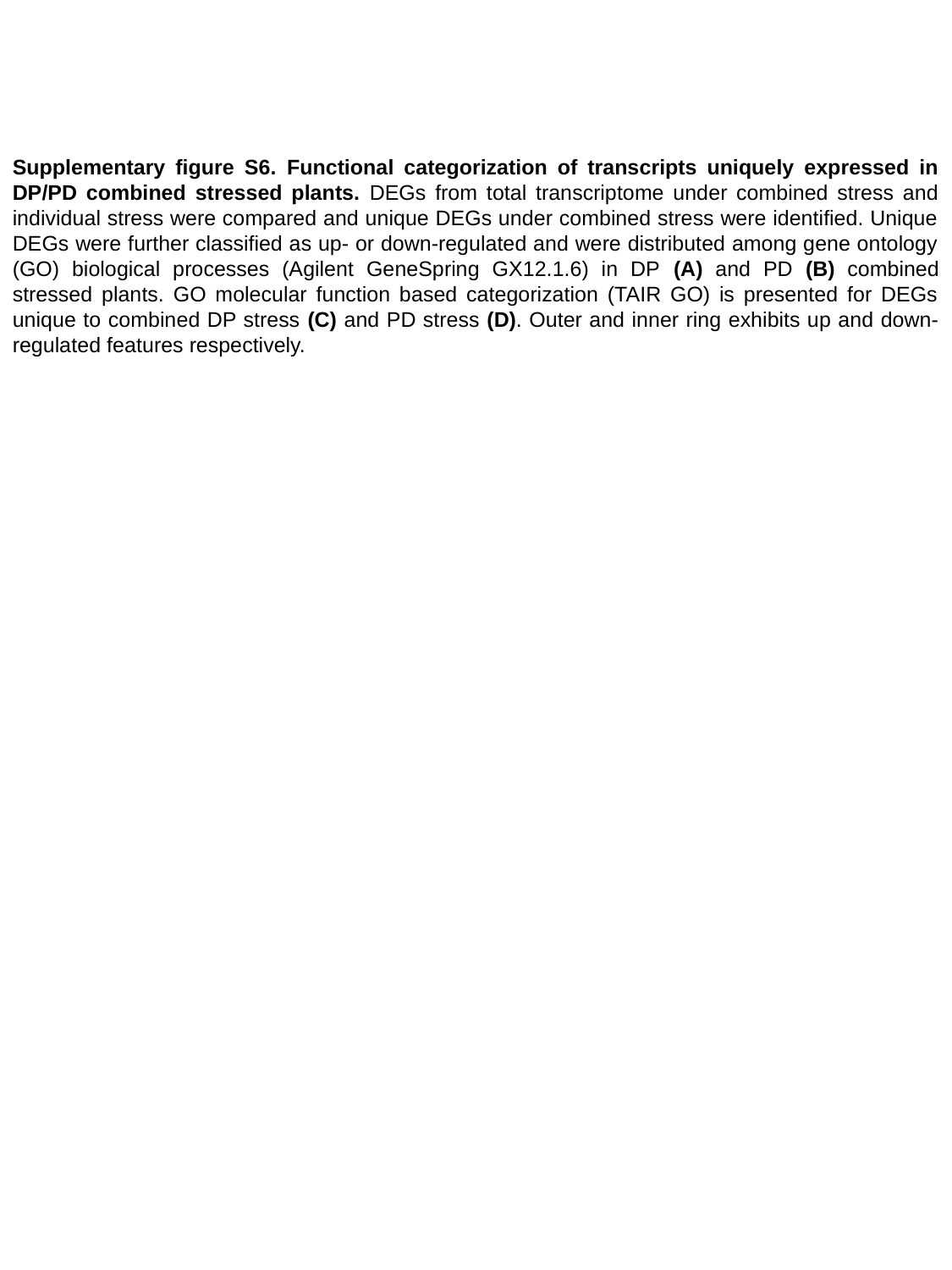

Supplementary figure S6. Functional categorization of transcripts uniquely expressed in DP/PD combined stressed plants. DEGs from total transcriptome under combined stress and individual stress were compared and unique DEGs under combined stress were identified. Unique DEGs were further classified as up- or down-regulated and were distributed among gene ontology (GO) biological processes (Agilent GeneSpring GX12.1.6) in DP (A) and PD (B) combined stressed plants. GO molecular function based categorization (TAIR GO) is presented for DEGs unique to combined DP stress (C) and PD stress (D). Outer and inner ring exhibits up and down-regulated features respectively.
