# Supplementary material for: Global Transcriptional Analysis Reveals Unique and Shared Responses in Arabidopsis thaliana Exposed to Combined Drought and Pathogen Stress
Source: Front Plant Sci. 2016 May 24;7:686. doi: 10.3389/fpls.2016.00686 (PMC4878317; doi:10.3389/fpls.2016.00686)
Supplement: Supplementary file 12 [file Presentation7.PPTX]

## Slide 1
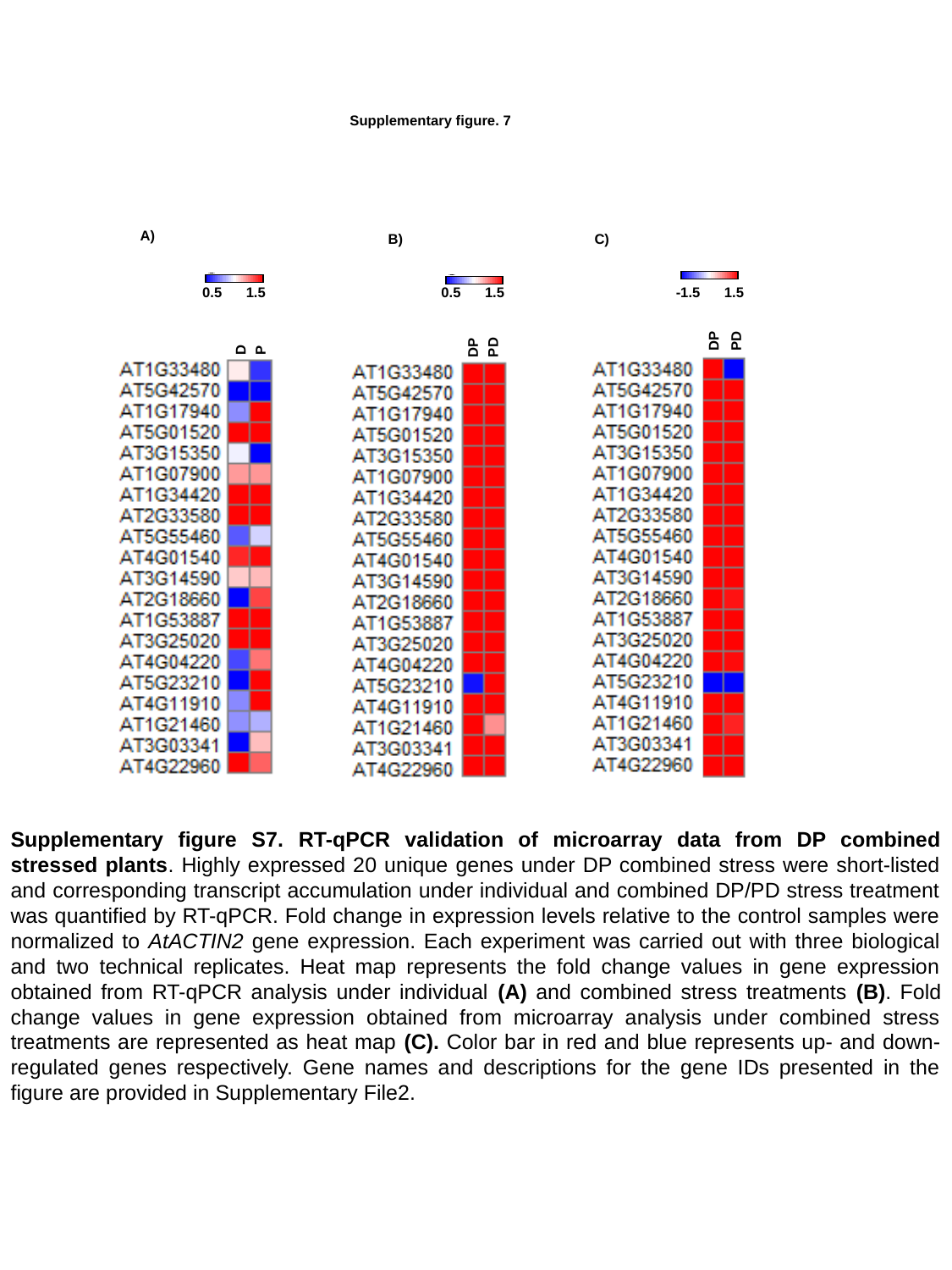

Supplementary figure. 7
A)
B)
C)
-1.5 1.5
0.5 1.5
D
P
0.5 1.5
PD
DP
PD
DP
Supplementary figure S7. RT-qPCR validation of microarray data from DP combined stressed plants. Highly expressed 20 unique genes under DP combined stress were short-listed and corresponding transcript accumulation under individual and combined DP/PD stress treatment was quantified by RT-qPCR. Fold change in expression levels relative to the control samples were normalized to AtACTIN2 gene expression. Each experiment was carried out with three biological and two technical replicates. Heat map represents the fold change values in gene expression obtained from RT-qPCR analysis under individual (A) and combined stress treatments (B). Fold change values in gene expression obtained from microarray analysis under combined stress treatments are represented as heat map (C). Color bar in red and blue represents up- and down-regulated genes respectively. Gene names and descriptions for the gene IDs presented in the figure are provided in Supplementary File2.
