# Supplementary material for: Global Transcriptional Analysis Reveals Unique and Shared Responses in Arabidopsis thaliana Exposed to Combined Drought and Pathogen Stress
Source: Front Plant Sci. 2016 May 24;7:686. doi: 10.3389/fpls.2016.00686 (PMC4878317; doi:10.3389/fpls.2016.00686)
Supplement: Supplementary file 13 [file Presentation8.PPTX]

## Slide 1
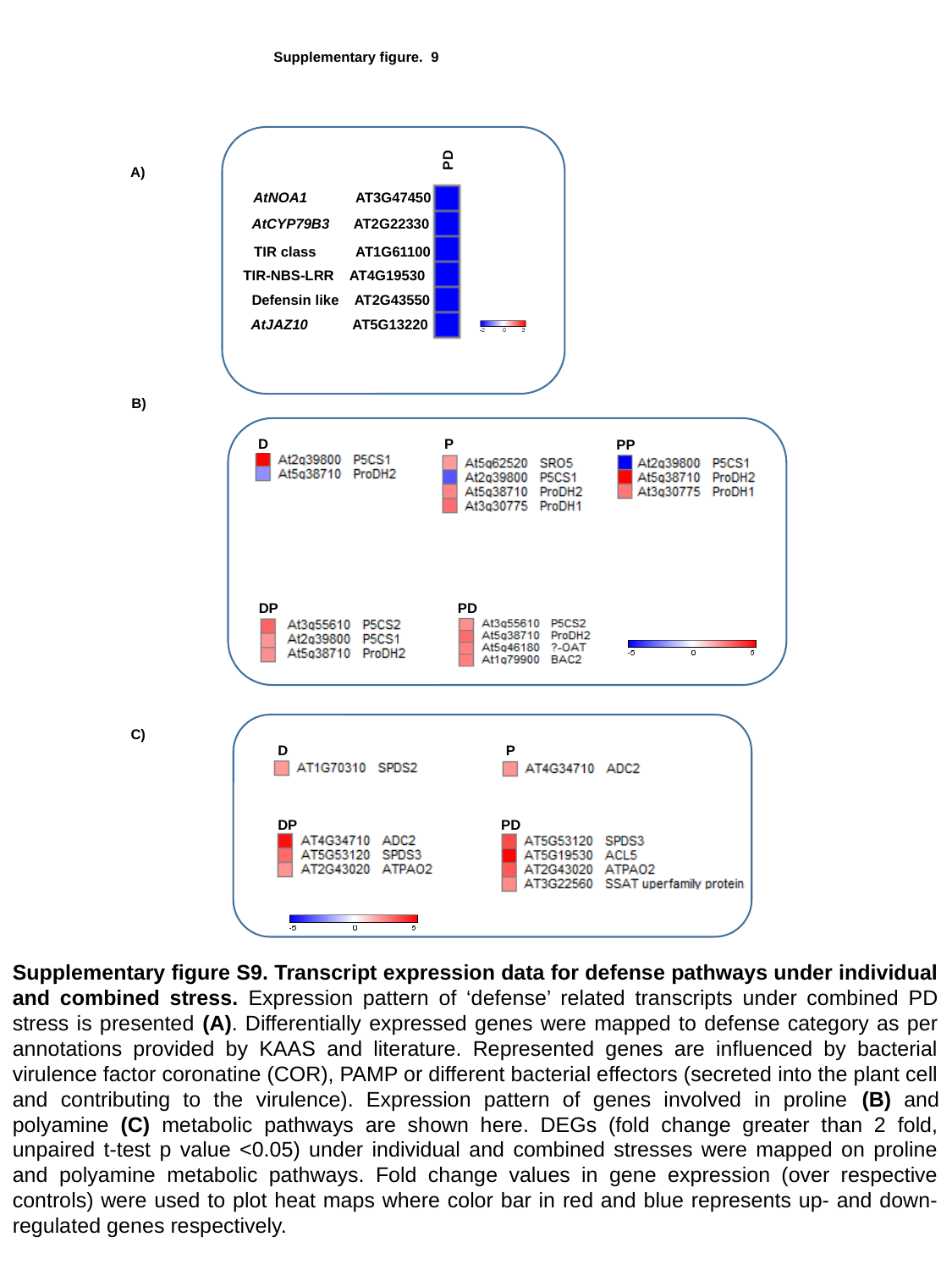

Supplementary figure. 9
PD
AtNOA1 AT3G47450
AtCYP79B3 AT2G22330
TIR class AT1G61100
TIR-NBS-LRR AT4G19530
Defensin like AT2G43550
AtJAZ10 AT5G13220
A)
B)
D
P
PP
DP
PD
D
P
DP
PD
C)
Supplementary figure S9. Transcript expression data for defense pathways under individual and combined stress. Expression pattern of ‘defense’ related transcripts under combined PD stress is presented (A). Differentially expressed genes were mapped to defense category as per annotations provided by KAAS and literature. Represented genes are influenced by bacterial virulence factor coronatine (COR), PAMP or different bacterial effectors (secreted into the plant cell and contributing to the virulence). Expression pattern of genes involved in proline (B) and polyamine (C) metabolic pathways are shown here. DEGs (fold change greater than 2 fold, unpaired t-test p value <0.05) under individual and combined stresses were mapped on proline and polyamine metabolic pathways. Fold change values in gene expression (over respective controls) were used to plot heat maps where color bar in red and blue represents up- and down-regulated genes respectively.
