# Supplementary material for: Global Transcriptional Analysis Reveals Unique and Shared Responses in Arabidopsis thaliana Exposed to Combined Drought and Pathogen Stress
Source: Front Plant Sci. 2016 May 24;7:686. doi: 10.3389/fpls.2016.00686 (PMC4878317; doi:10.3389/fpls.2016.00686)
Supplement: Supplementary file 15 [file Presentation10.PPTX]

## Slide 1
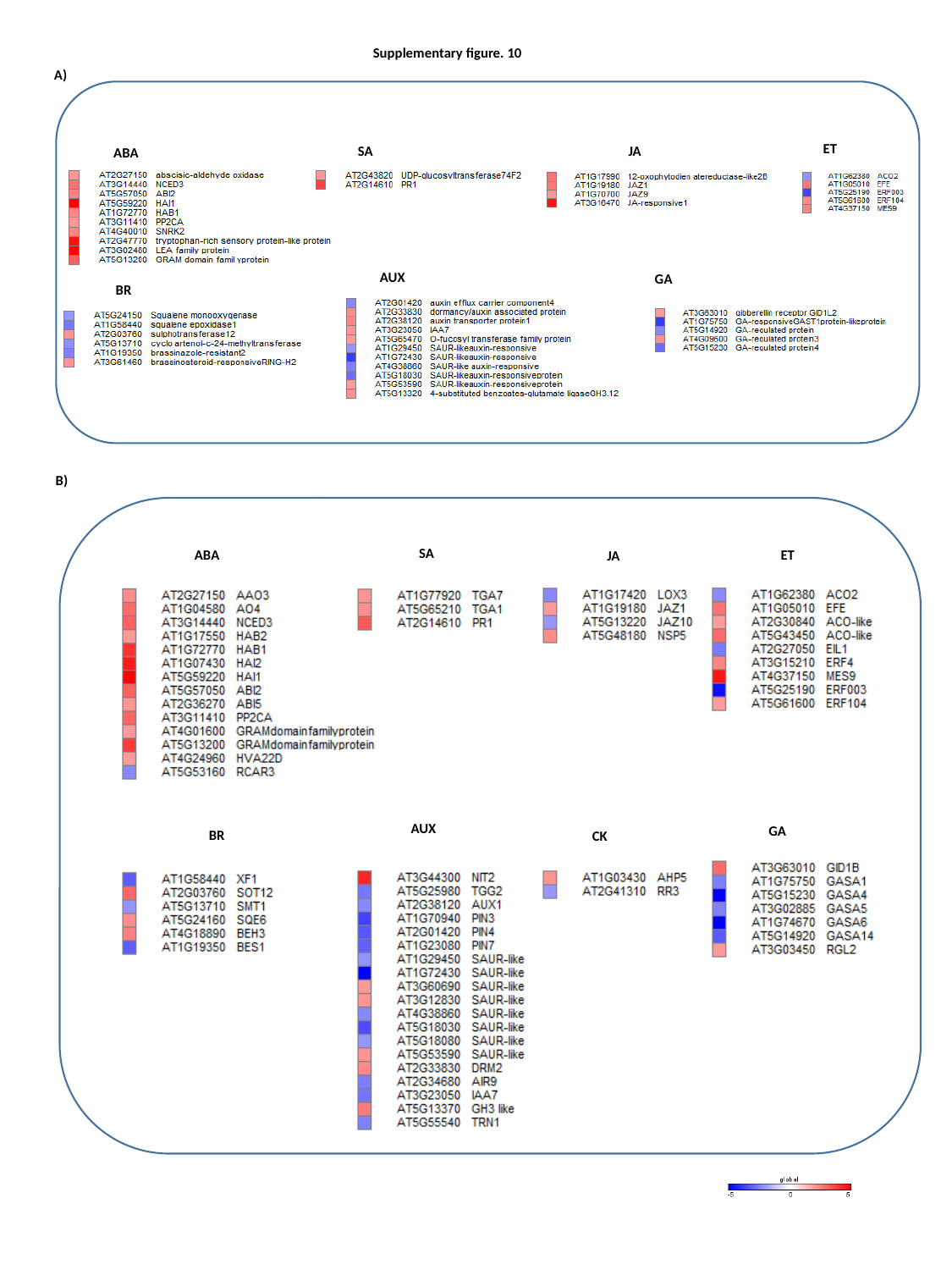

Supplementary figure. 10
A)
ET
JA
SA
ABA
AUX
GA
BR
B)
SA
ABA
ET
JA
AUX
GA
BR
CK

## Slide 2
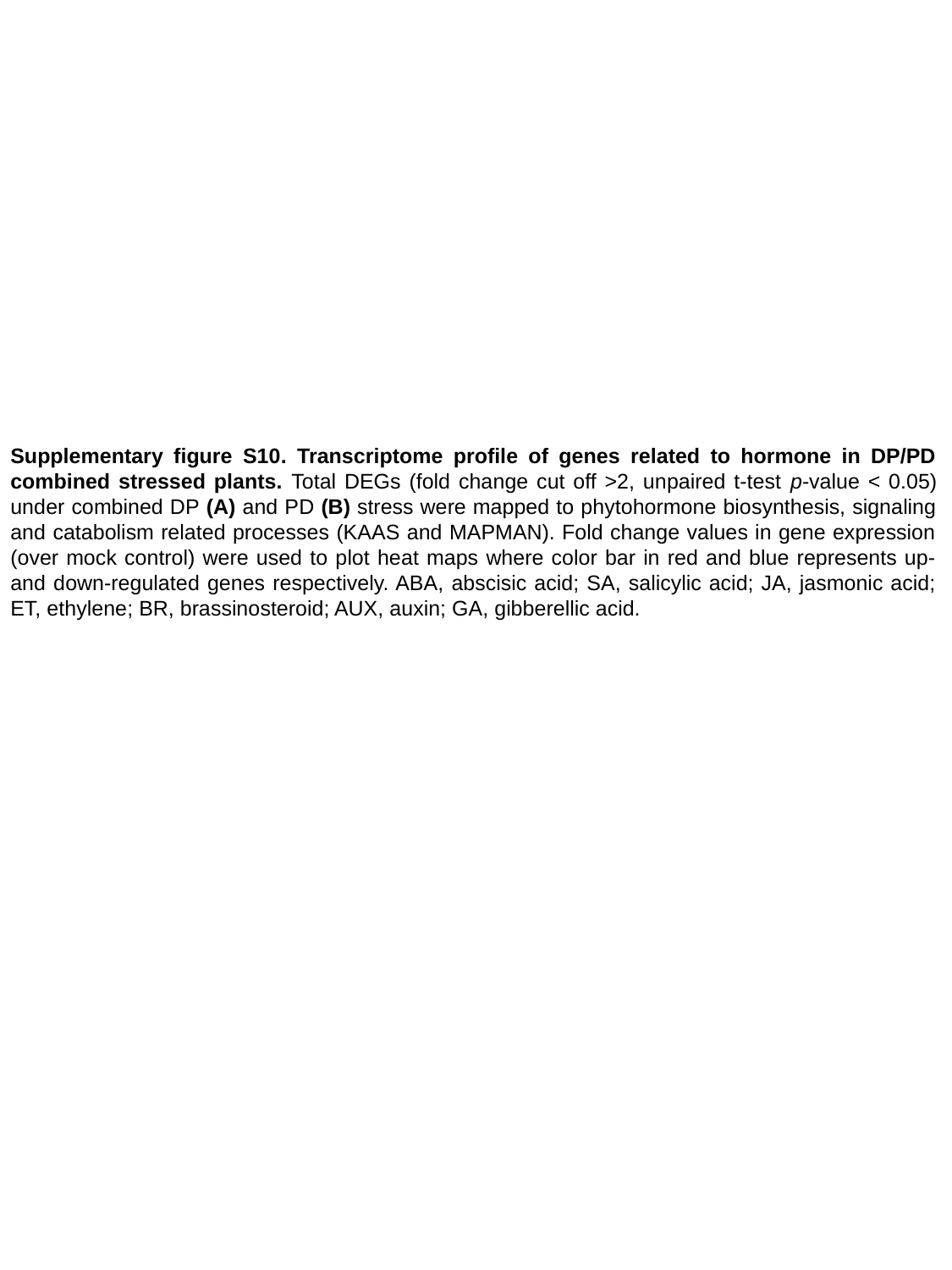

Supplementary figure S10. Transcriptome profile of genes related to hormone in DP/PD combined stressed plants. Total DEGs (fold change cut off >2, unpaired t-test p-value < 0.05) under combined DP (A) and PD (B) stress were mapped to phytohormone biosynthesis, signaling and catabolism related processes (KAAS and MAPMAN). Fold change values in gene expression (over mock control) were used to plot heat maps where color bar in red and blue represents up- and down-regulated genes respectively. ABA, abscisic acid; SA, salicylic acid; JA, jasmonic acid; ET, ethylene; BR, brassinosteroid; AUX, auxin; GA, gibberellic acid.
